# Region 4 of *Rhizobium etli* Primary Sigma Factor (SigA) Confers Transcriptional Laxity in *Escherichia coli*

**DOI:** 10.3389/fmicb.2016.01078

**Published:** 2016-07-13

**Authors:** Orlando Santillán, Miguel A. Ramírez-Romero, Luis Lozano, Alberto Checa, Sergio M. Encarnación, Guillermo Dávila

**Affiliations:** ^1^Programa de Genómica Evolutiva, Centro de Ciencias Genómicas, Universidad Nacional Autónoma de MéxicoCuernavaca, Mexico; ^2^Oncomedic IncorporationCiudad Cuauhtémoc, Mexico; ^3^Programa de Genómica Funcional de Procariontes, Centro de Ciencias Genómicas, Universidad Nacional Autónoma de MéxicoCuernavaca, Mexico; ^4^Laboratorio Internacional de Investigación sobre el Genoma Humano, Universidad Nacional Autónoma de MéxicoJuriquilla, Mexico

**Keywords:** *Rhizobium etli*, transcriptional laxity, primary sigma factor, SigA, RpoD, region 4, lax consensus promoter, housekeeping

## Abstract

Sigma factors are RNA polymerase subunits engaged in promoter recognition and DNA strand separation during transcription initiation in bacteria. Primary sigma factors are responsible for the expression of *housekeeping* genes and are essential for survival. RpoD, the primary sigma factor of *Escherichia coli*, a γ-proteobacteria, recognizes consensus promoter sequences highly similar to those of some α-proteobacteria species. Despite this resemblance, RpoD is unable to sustain transcription from most of the α-proteobacterial promoters tested so far. In contrast, we have found that SigA, the primary sigma factor of *Rhizobium etli*, an α-proteobacteria, is able to transcribe *E. coli* promoters, although it exhibits only 48% identity (98% coverage) to RpoD. We have called this the *transcriptional laxity phenomenon*. Here, we show that SigA partially complements the thermo-sensitive deficiency of RpoD285 from *E*. *coli* strain UQ285 and that the SigA region σ4 is responsible for this phenotype. Sixteen out of 74 residues (21.6%) within region σ4 are variable between RpoD and SigA. Mutating these residues significantly improves SigA ability to complement *E*. *coli* UQ285. Only six of these residues fall into positions already known to interact with promoter DNA and to comprise a helix-turn-helix motif. The remaining variable positions are located on previously unexplored sites inside region σ4, specifically into the first two α-helices of the region. Neither of the variable positions confined to these helices seem to interact directly with promoter sequence; instead, we adduce that these residues participate allosterically by contributing to correct region folding and/or positioning of the HTH motif. We propose that transcriptional laxity is a mechanism for ensuring transcription in spite of naturally occurring mutations from endogenous promoters and/or horizontally transferred DNA sequences, allowing survival and fast environmental adaptation of α-proteobacteria.

## Introduction

The bacterial DNA-dependent RNA polymerase (RNAP) holoenzyme (Eσ) consists of a core enzyme (subunits $\alpha_{2}\beta \beta^{\prime }\omega$; E) and one sigma factor (σ) subunit, which recognizes DNA promoters to initiate sequence-specific transcription (Lee et al., 2012). During transcription, sigma factors provide the most fundamental mechanism for orchestrating broad changes in the gene expression profile, making them key proteins during this process (Wösten, 1998).

Based on amino acid sequence and structure, sigma factors are divided into two main families: σ^70^ and σ^54^, respectively. Numerical super indexes denote their molecular weight (in kDa) based on data from *Escherichia coli*, a γ-proteobacteria (Gruber and Gross, 2003). The σ^70^ family is subdivided into four groups. Group 1 comprises all known primary sigma factors (also known as RpoD, housekeeping-σ, σ^D^ or σ^70^). Groups 2 through 4 comprise the alternative sigma factors, which are involved in transcribing specialized regulons, i.e., stationary-phase, heat-shock, extra cytoplasmic-stress, nitrogen-metabolism, or flagellar synthesis (Gruber and Gross, 2003). *In vivo*, each sigma factor recognizes a different non-overlapping set of promoter sequences (Gruber and Gross, 2003). In *E. coli*, the promoter consensus sequence recognized by RpoD is: 5′- TTGACA (–35 box)—spacer (17 ± 2 bp)—TATAAT (–10 box)—3′. The box names indicate their relative positions from the transcription start site (also called +1; Hawley and McClure, 1983; Harley and Reynolds, 1987; Shultzaberger et al., 2007; Shimada et al., 2014).

Group 1 sigma factors have a modular composition made up of four conserved (σ1, σ2, σ3, and σ4) and one variable (σNCR, non-conserved region) regions. Every region of *E. coli* RpoD has been assigned to at least one function, e.g., σ1 is involved in autoinhibition of free sigma (together with σ4) and participates in promoter binding and escape (Dombroski et al., 1992; Wilson and Dombroski, 1997; Baldwin and Dombroski, 2001; Camarero et al., 2002; Haugen et al., 2006; Bochkareva and Zenkin, 2013); σNCR helps promoter escape and inhibits RpoD-dependent transcription pausing (Baldwin et al., 2002; Gruber and Gross, 2003; Leibman and Hochschild, 2007); σ2 participates in E binding, DNA melting and promoter recognition of the –10 box (Lesley and Burgess, 1989; Waldburger et al., 1990; Huang et al., 1997; Panaghie et al., 2000; Liao et al., 2002; Schroeder et al., 2007; Feklistov and Darst, 2011); σ3 plays a role in extended –10 promoter recognition, binding to the initiating nucleotide, the production of abortive RNA (Hernandez et al., 1996; Murakami, 2002) and promoter escape (Kulbachinskiy and Mustaev, 2006) and region σ4 aids in recognition of the promoters –35 box and interaction with transcriptional regulators such as 6S RNA (Gardella et al., 1989; Camarero et al., 2002; Dove et al., 2003; Klocko and Wassarman, 2009).

Almost every sigma region contains a tract that interacts with E (Nagai and Shimamoto, 1997; Murakami, 2002; Schroeder et al., 2007). Among the seven distinct sigma factors of *E. coli*, RpoD has the highest affinity for E (Maeda et al., 2000).

*Rhizobium etli*, an α-proteobacteria, can be found as a free living soil organism or as a symbiont in root nitrogen-fixing nodules of *Phaseolus vulgaris* (common bean). The whole genomic sequence of *R. etli* CFN42 consists of a circular chromosome and six large plasmids, with an average G+C content of 61.5% (González et al., 2006). The α-proteobacteria class encompasses not only a wide variety of lifestyles but also broad genome sizes. Many of their members show a multireplicon genome structure (Capela et al., 2001; Galibert et al., 2001; Mackenzie et al., 2001; Wood et al., 2001; González et al., 2006; Strnad et al., 2010). Additionally, *R. etli* contains a large number of sigma factors (one primary sigma gene, two copies of *rpoH*, two copies of *rpoN*, and 18 genes of the extracytoplasmic factor group), a feature shared with other nitrogen-fixing organisms, like *Bradyrhizobium japonicum, Mesorhizobium loti*, and *Sinorhizobium meliloti* (Mittenhuber, 2002).

The *R. etli* primary sigma factor gene (*sigA*) encodes a protein of 685 amino acid residues with a molecular weight of 77.18 kDa. The amino acid sequence of SigA shows 48% identity (98% coverage) to RpoD. Like other α-proteobacteria (*S. meliloti, Caulobacter crescentus, Rhodobacter sphaeroides, Rhodobacter capsulatus*), *R. etli* primary sigma factor is capable of transcribing most of the RpoD-dependent promoters tested so far (transcriptional laxity). On the other hand, *R. etli, S. meliloti, C. crescentus, R. sphaeroides*, and *R. capsulatus* primary sigma dependent promoters are poorly or not transcribed at all by RpoD (Karls et al., 1993; Malakooti et al., 1995; Cullen et al., 1997; MacLellan et al., 2006; Ramírez-Romero et al., 2006), which suggests some differences between the *E. coli* and the α-proteobacterial transcriptional machineries, perhaps at the level of promoter recognition by the primary sigma factor.

The characterization of the transcriptional molecular basis in organisms with agricultural importance like *R. etli* is fundamental, because it could provide information for future biotechnological applications. Among the potential applications are: heterologous expression of genes contributing to enhance symbiosis or nitrogen fixation, design of promoters that ensure transcription among other symbiotic α-proteobacteria and engineering of sigma factors to gain broad transcriptional capacities. We chose *R*. *etli* SigA gene as a model of transcriptional laxity in α-proteobacteria.

To identify SigA regions involved in transcriptional laxity we made a library of chimeric genes exchanging the regions of RpoD and SigA. We constructed 14 non-redundant possible combinations between both sigma factors. We then tested their ability to complement the thermo-sensitive deficiency of RpoD285 primary sigma factor of *E*. *coli* strain UQ285 (*E. coli rpoD285*; Harris et al., 1978; Hu and Gross, 1983). The results show that whenever SigA region σ4 is present, the carrier chimera is able to sustain growth of *E*. *coli rpoD285* at restrictive temperature (42°C). Mutating residues at variable positions within the first two α-helices enhances the ability of SigA to complement the *E. coli rpoD285* phenotype. We propose that these helices participate in correct folding and positioning of the HTH motif found on region σ4. The HTH motif is responsible for promoter recognition.

## Materials and methods

### Bacterial strains and plasmids

Relevant information about bacterial strains and plasmids is listed in Table 1.

**Table 1:** **Bacterial strains and plasmids**.

**Name**	**Growth temperature**	**Relevant features**
*E. coli* BW28465	37°C	Chromosomal deletion of *rpoS* gene. No antibiotic resistance gene present. Obtained from Coli Genetic Stock Center, Yale University, New Haven, USA (Zhou et al., 2003).
*E. coli* CAG1	30–42°C	Chromosomal encoded thermo-sensitive RpoD allele (*rpoD*800). Streptomycin resistant. Obtained from Coli Genetic Stock Center, Yale University, New Haven, USA (Liebke et al., 1980).
*E. coli* DH5α	30–37°C	Plasmid propagation and DNA purification. Host strain for pUC19PnRFP library experiments. Nalidixic acid resistant.
*E. coli* S17	30–37°C	Donor strain used for conjugation with *R. etli* CF42. Spectinomycin resistant. Nalidixic acid sensitive.
*E. coli* UQ285	30–42°C	Chromosomal encoded thermo-sensitive RpoD allele (*rpoD*285). No antibiotic resistance gene present. Obtained from Coli Genetic Stock Center, Yale University, New Haven, USA (Harris et al., 1978).
*R. etli* CFN42	30°C	Template for amplification of wild type sigA gene. Host strain for the pBBR1MCS5PnRFP library. Nalidixic acid resistant (González et al., 2006).
pBBR1MCS5	NA	GenBank GI: 833825. Length: 4768 bp. Parental vector for PnRFP library. Gentamicin resistance.
pBBR1MCS5PrpoDconsRFP	NA	RpoD promoter consensus sequence controlling transcription of RFP gene. Gentamicin resistance.
pBBR1MCS5PsigAconsRFP	NA	SigA promoter consensus sequence controlling transcription of RFP gene. Gentamicin resistance.
pBBR1MCS5PlessRFP	NA	Promoter-less RFP construction. Negative control. Gentamicin resistance.
pUC19	NA	GenBank GI: 6691170. Length: 2686 bp. Parental vector for PnRFP library. Ampicillin resistance.
pUC19PrpoDconsRFP	NA	RpoD promoter consensus sequence controlling transcription of RFP gene. Ampicillin resistance.
pUC19PsigAconsRFP	NA	SigA promoter consensus sequence controlling transcription of RFP gene. Ampicillin resistance.
pUC19PlessRFP	NA	Promoter-less RFP construction. Negative control. Ampicillin resistance
pUC19*rpoD*	NA	Wild type *rpoD* gene for construction of chimera01–02. Ampicillin resistance
pUC19*sigA*	NA	Wild type *sigA* gene for construction of chimera01–02. Ampicillin resistance
pRK415	NA	GenBank GI: 130693907. Length: 10690 bp. Parental vector for sigma library. Tetracycline resistance
pRK415*rpoD*	NA	Wild type *rpoD* gene. Positive control for UQ285 complementation experiments. Tetracycline resistance
pRK415*sigA*	NA	Wild type *sigA* gene. UQ285 complementation experiments. Tetracycline resistance
pRK415*chim01–14*	NA	Chimeric gene library. UQ285 complementation experiments. Tetracycline resistance
pRK415*sigAmut01–03*	NA	sigA region4 mutant library. UQ285 complementation experiments. Tetracycline resistance

*NA, not applicable*.

### Bacterial growth and transformation conditions

All *E. coli* strains were grown aerobically in Luria-Bertani (LB) medium supplemented with the appropriate antibiotics. *E. coli* DH5α and S17 strains (used for plasmid propagation or donation) were grown at 37°C. *E. coli rpoD*285 strain was grown at 30°C (permissive temperature) or 42°C (restrictive temperature). *R. etli* CFN42 was grown aerobically at 30°C in Peptone-Yeast (PY) medium supplemented with 7 mM CaCl_2_. Antibiotics were added at the following final concentrations (μg ml^−1^): ampicillin (Amp) 100, gentamycin (Gen) 20, nalidixic acid (Nal) 20, and tetracycline (Tet) 10. When necessary, isopropyl-β-D-thiogalactopyranoide (IPTG) was added to a final concentration of 0.5 mM. Bacterial transformation by electroporation was carried out at 1.8 V, 200 Ω, and 25 μF on 0.1 cm sterile disposable cuvettes (BioRad).

### Wild type primary sigma factor genes amplification and cloning

Total DNA was purified from *E*. *coli* DH5α and *R*. *etli* CFN42 strains, respectively (Sambrook and Russell, 2001). The forward oligonucleotide sequence included an *Xba*I recognition site and an optimal ribosome binding site (RBS), 5′-AGGAGA-3′, six base pairs away from the start codon. The reverse oligo contained a *Kpn*I recognition sequence. Oligonucleotides were designed to amplify only the coding sequence of the corresponding primary sigma factor genes. Once amplified and purified, wild type genes were digested with *Kpn*I-*Xba*I and cloned into pRK415. The pRK415 cloned fragments are under the control of the *E*. *coli* lactose promoter (*Plac*). Transformants were selected on LB/Tet plates grown at 37°C. The oligonucleotides described above were also used in the last amplification step for the production of a chimeric library. Consequently, all constructs involving the expression vector pRK415 share the same RBS sequence and cloning sites. First members of the library were pRK415*rpoD* (pRK*rpoD*) and pRK415*sigA* (pRK*sigA*). Wild type genes were amplified using Platinum *Taq* High Fidelity DNA polymerase (Invitrogen). Restriction enzymes were obtained from New England Biolabs (NEB). All primer sequences are listed in Table 2.

**Table 2:** **Oligonucleotide sequences**.

**No**.	**Name**	**Sequence**	**Length (nt)**	**Relevant features**	
1	r1Eco-FWD	GCTCTAGAGA**AGGAGA**TATCATATGGAGCAAAACCCGCAGTCA	43	*XbaI* site; wild type gene amplification and pRK415 cloning	
2	r4Eco-REV	GGGGTACCTTAATCGTCCAGGAAGCTACG	29	*KpnI* site; wild type gene amplification and pRK415 cloning	
3	r1Ret-FWD	GCTCTAGAGA**AGGAGA**TATCATATGGCAACCAAGGTCAAAGAG	43	*XbaI* site; wild type gene amplification and pRK415 cloning	
4	r4Ret-REV	GGGGTACCTTAGCTGTCCAGAAAGCTTCT	29	*KpnI* site; wild type gene amplification and pRK415 cloning	
5	Amp-FWD	ACACTAGTGAAAGTAAAAGAT	21	*SpeI* site inserted, synonym mutation. Construction of chim01 and 02	
6	Amp-REV	TCACTAGTGTTTCTGGGTGAG	21	*SpeI* site inserted, synonym mutation. Construction of chim01 and 02	
7	r2VosEco-FWD	AACTTAAGGCTGGTTATTTCTATCGCT	27	*AflII* site inserted, synonym mutation. Construction of chim01–02	
8	r2VosEco-REV	GCCTTAAGTTCGCTTCAACCATCTCTT	27	*AflII* site inserted, synonym mutation. Construction of chim01–02	
9	r2VosRet-FWD	AACTTAAGGCTCGTCATTTCAATCGCC	27	*AflII* site inserted, synonym mutation. Construction of chim01–02.	
10	r2VosRet-REV	GCCTTAAGTTCGCTTCGACCATTTCCT	27	*AflII* site inserted, synonym mutation. Construction of chim01–02	
11	r1Eco/r2Ret-FWD	CGCTAAGCGTATTGAAGACGGGATCGAGACGATGATCGCCGGCC	44	Internal oligo for chimeric gene assembly of chim05–14	
12	r1Eco/r2Ret-REV	CACAGAGGCCGGCGATCATCGTCTCGATCCCGTCTTCAATACGC	44	Internal oligo for chimeric gene assembly of chim05–14	
13	r2Eco/r3Ret-FWD	CAGGCGCGCACCATCCGTATTCCGGTGCACATGATCGAGACG	42	Internal oligo for chimeric gene assembly of chim05–14	
14	r2Eco/r3Ret-REV	CGTCTCGATCATGTGCACCGGAATACGGATGGTGCGCGCCTG	42	Internal oligo for chimeric gene assembly of chim05–14	
15	r3Eco/r4Ret-FWD	GGATTCTGCGACCACCGAAAGCCTGCGCGAGACGACGACCCGCG	44	Internal oligo for chimeric gene assembly of chim05–14	
16	r3Eco/r4Ret-REV	AAAACGCGGGTCGTCGTCTCGCGCAGGCTTTCGGTGGTCGCAGAATC	47	Internal oligo for chimeric gene assembly of chim05–14	
17	r1Ret/r2Eco-FWD	AAGCGCATCGAAGCCGGCCGCAACCAGGTTCAATGCTCCGTTG	43	Internal oligo for chimeric gene assembly of chim05–14	
18	r1Ret/r2Eco-REV	GCAACGGAGCATTGAACCTGGTTGCGGCCGGCTTCGATGCGCTTAG	46	Internal oligo for chimeric gene assembly of chim05–14	
19	r2Ret/r3Eco-FWD	CGCCGACCAGGCCCGCACGATCCGCATTCCGGTGCATATGATTGAGACC	49	Internal oligo for chimeric gene assembly of chim05–14	
20	r2Ret/r3Eco-REV	GATGGTCTCAATCATATGCACCGGAATGCGGATCGTGCGGGCCTGG	46	Internal oligo for chimeric gene assembly of chim05–14	
21	r3Ret/r4Eco-FWD	CGACGCCGCCATCCAGGCGAACCTGCGTGCGGCAACGCACGAC	43	Internal oligo for chimeric gene assembly of chim05–14	
22	r3Ret/r4Eco-REV	CGTGCGTTGCCGCACGCAGGTTCGCCTGGATGGCGGCGTC	40	Internal oligo for chimeric gene assembly of chim05–14	
23	UNIpBBR-FWD	ACCCGGGATCCCGATCGTAATCCTCTCGCCGACGC	35	*SmaI* site; For PnRFP amplification and cloning into pBBR1MCS5	
24	RFPpBBR-REV	AGGGCCCATCGATGTATATAAACGC	25	*ApaI* site; For PnRFP amplification and cloning into pBBR1MCS5	
25	UNIpUC-FWD	AAAGGATCCCGATCGTAATCCTCTCGCC	28	*BamHI* site; For PnRFP amplification and cloning into pUC19	
26	RFPpUC-REV	AAAGCATGCGTATATAAACGCAGAAAGG	28	*SphI* site; For PnRFP amplification and cloning into pUC19	
27	RFP-FWD	GATCTGAATTCGAAAG**AGGAGA**AATACTAGATGGC	35	*EcoRI* site; For RFP amplification and ligation to promoter fragment	
28	PrpoD-REV	GATTTGAATTCgctagc**ATTATA**CCTAGGACTGAgctagc**TGTCAA**GGCTGCTGGTCGAGAGCTTC	66	*EcoRI*,*NheI* sites; Promoter amplification and ligation to RFP fragment	
29	PsigA-REV	GATTTGAATTC**ATATAG**CCTAGGACTGAgctagc**GTCAAG**GGCTGCTGGTCGAGAGCTTC	60	*EcoRI* site; Promoter amplification and ligation to RFP fragment	

*Restriction enzyme sites are underlined, except for NheI sites that appear in lowercase. RBS are in bold. Eco denotes oligonucleotides for RpoD; Ret, oligonucleotides for SigA. The –35 and –10 boxes of promoter consensuses appear in underlined bold. FWD, forward primer, REV, reverse primer, Vos, plasmid recovery technique for assembling chimeras, Amp, ampicillin resistance gene primers, UNI, universal primer, pBBR, primers for cloning into pBBR1MCS5 vector, pUC, primers for cloning into pUC19 vector, RFP, red fluorescent protein gene primers*.

### *In silico* oligonucleotide design and chimeric gene assembly

**Figure 1:**
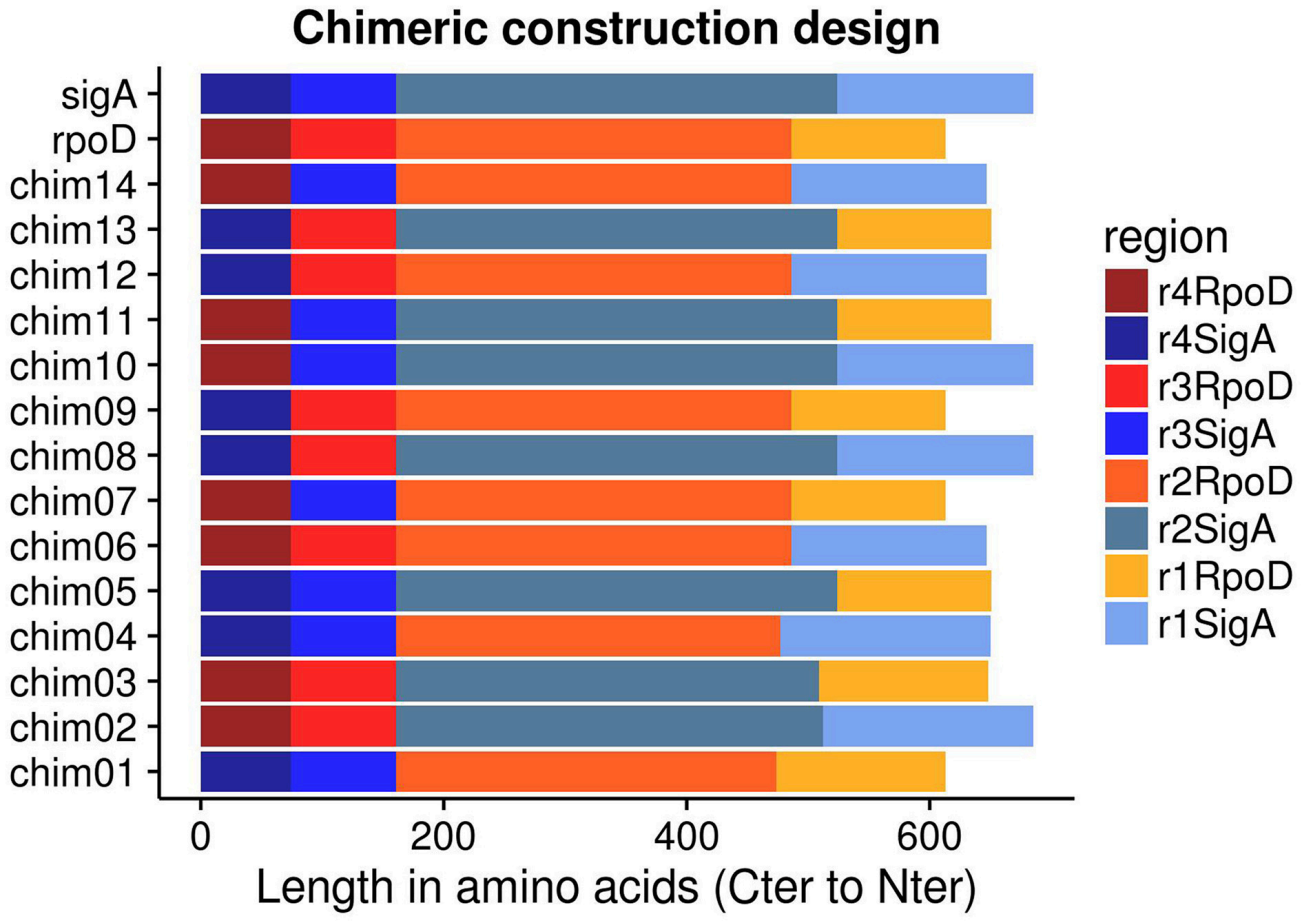
**pRK415sigma library members were arranged in Carboxy-terminal to Amino-terminal orientation because regions σ3 and σ4 have the same length between RpoD and SigA**.

Amino acid and nucleotide sequences of *E. coli rpoD* and *R. etli sigA* were obtained using Artemis Genome Browser (release 15.0.0, RRID:SCRRRID:SCR_004267; Rutherford et al., 2000) from complete chromosome sequences of *E. coli* strain K12 substrain MG1655 (GenBank: NC_000913) and *R. etli* CFN42 (NC_007761), respectively. SigA region identification was done in accordance to RpoD amino acid sequence (Gruber and Gross, 2003) and oligonucleotide design corresponded to those boundaries. Fourteen non-redundant chimeric genes were created, shuffling regions between the two wild type genes (Figure 1). Resembling functional primary sigma factors, each chimeric gene included four regions (σ1-σ2-σ3-σ4). σNCR and σ2 constitute domain 2 in RpoD (Gruber and Gross, 2003); for that reason, these two regions were considered as one and designated by σ2 alone. Sequence alignments were performed using MUSCLE (version 3.8.31, RRID:SCRRRID:SCR_011812; Edgar, 2004). Chimeric genes were assembled using *ad hoc* scripts written in R language (version 2.15.1; R Development Core Team, 2008, RRID:SCRRRID:SCR_001905) and subsequently manually verified. All programs mentioned above were run locally.

### Construction of chimeric genes

Chimeric sequences were assembled by three different approaches: overlapping PCR products, plasmid recovery (modified from Vos and Kampinga, 2008) and gene synthesis. Fourteen different chimeric genes were obtained, according to the *in silico* design. All chimeric, wild type genes and *sigA* mutants were cloned into pRK415 (Keen et al., 1988; Hülter and Wackernagel, 2008) using *Kpn*I-*Xba*I restriction enzymes (pRK415sigma library). Platinum *Taq* High Fidelity DNA polymerase was used for all amplification reactions. Restriction enzymes were obtained from NEB.

### Chimera assembly by modified plasmid recovery technique

The two wild type genes, *rpoD* and *sigA*, were cloned independently in vector pUC19 (Norrander et al., 1983) between *Xba*I and *Kpn*I restriction sites. These constructs, pUC*rpoD* and pUC*sigA*, were used as DNA templates. Oligonucleotide sequences were designed to obtain two complementary segments of each construct, disrupting the ampicillin resistance gene (present on the vector) and the sigma factor gene (on the desired target region; Vos and Kampinga, 2008). After performing amplification reactions, *DpnI* digestion of the template DNA and gel purification of the corresponding bands, we ended up with four fragments: pUC19rpoDσ1-σ2_partA, pUC19rpoDσ3-σ4_partB, pUC19sigAσ1-σ2_partA, and pUC19sigAσ3-σ4_partB. Each DNA fraction was digested with *Afl*II-*Spe*I restriction enzymes, then, complementary DNA fragments were mixed (e.g., chimera01: pUC19rpoDσ1-σ2_partA and pUC19sigAσ3-σ4_partB) and ligated overnight at 16°C. The ligation reaction was used to transform DH5α cells and transformants were selected on LB/Amp plates at 37°C. After verifying them by PCR and re-digestion with *Kpn*I-*Xba*I enzymes, candidate constructs (pUC*ch01* and pUC*ch02*) were used as templates for another round of amplification with external oligonucleotides. Finally, *Kpn*I-*Xba*I digestion and cloning into pRK415 were done with these fragments, producing the constructs pRK415*chim01* (pRK*ch01*) and pRK415*chim02* (pRK*ch02*). Transformants were selected on LB/Tet plates at 37°C. DNA fragments and final products were amplified using Phusion High Fidelity DNA polymerase, which yields blunt-ended products only. DNA polymerase, ligase, and restriction enzymes were obtained from NEB.

### Chimera assembly by gene synthesis

The DNA sequences of chimeras 03 and 04 were assembled *in silico* according to protein region delimitation based on RpoD (Gruber and Gross, 2003) and secondary structure prediction results from the psipred server (Buchan et al., 2013 RRID:SCRRRID:SCR_010246). These chimeras were synthesized by GeneArt^TM^ Gene Synthesis (ThermoFisher Scientific). Synthetic chimeric gene sequences were re-amplified using Platinum *Taq* High Fidelity DNA polymerase, digested with *Kpn*I-*Xba*I and cloned into pRK415. In this way, we obtained the constructs pRK415*chim03* (pRK*ch03*) and pRK415*chim04* (pRK*ch04*). DH5α transformants were selected on LB/Tet plates at 37°C.

### Chimera assembly by overlapping PCR products

Primers used for this technique required two important features: target site amplification and overlap sequences. Each part has a minimum length of 20 base pairs (bp). Oligonucleotide design considered the 3′-A overhang ends produced by Platinum *Taq* High Fidelity DNA polymerase (Table 2). In order to fuse two DNA segments, we proceeded as follows: (1) amplify each fragment independently by PCR, (2) purify each band from agarose gel (Purification kit, Roche), (3) mix the purified fragments into the assembly PCR, where the overlapping region will produce the 3′-OH DNA end required for polymerization, (4) make an enrichment reaction using external oligonucleotides, and (5) purify the assembled DNA from an agarose gel. After purification, the DNA was digested with *Kpn*I-*Xba*I restriction enzymes, cloned into pRK415 and transformed into DH5α. Transformants were selected on LB/Tet plates at 37°C. Chimeras 05 through 14 were assembled by this method, yielding the constructs pRK415*chim05* to *chim14* (pRK*ch05-ch14*).

### SigA mutant constructs

An amino acid sequence alignment between RpoD and SigA genes showed that they differ in 16 out of the 74 residues along region σ4. SigA residues were replaced by their corresponding RpoD counterparts (changing corresponding codons) at these variable positions. Three *sigA* mutants were obtained with an average of five amino acid substitutions each. After the *in silico* mutagenesis, the resulting sequences and the pRK415 plasmid DNA were sent to GenScript (NJ, USA). GenScript synthesized, sequenced and cloned mutant sequences into pRK415 (pRK*sigAm01, sigAm02*, and *sigAm03*).

### RFP reporter gene constructs

Three promoters (RpoD and SigA consensuses and a promoter-less sequence) were fused with the Red Fluorescent Protein gene independently (*P*_*n*_RFP). *Pn*RFP constructs were cloned into two different vectors, pBBR1MCS5 (Kovach et al., 1995) and pUC19 (Norrander et al., 1983). We constructed pBBR1MCS5 set first. Consensus promoter sequences of RpoD (*P*_*Eco*_; Hawley and McClure, 1983; Harley and Reynolds, 1987; Shultzaberger et al., 2007; Shimada et al., 2014) and SigA (*P*_*Ret*_; Ramírez-Romero et al., 2006) were introduced in the reverse oligonucleotide and a promoter-less intergenic region (pD00022 gene) of the *R. etli* genome was used as template for the amplification reaction (this sequence ended upstream of the promoter). Separately, the RFP coding sequence flanked by an upstream RBS site (5′-AGGAGA-3′) and a downstream strong transcriptional terminator was amplified by PCR from plasmid pJ61002 (iGem repository). These two DNA fragments were digested with *Eco*RI and ligated with T4 DNA ligase (NEB). After ligation, another round of DNA amplification was carried out using the external oligonucleotides. Constructs were cloned into pBBR1MCS5 between *Sma*I and *Apa*I sites (inside multiple cloning site, MCS). Insert orientation was chosen in order to minimize the effect of outer promoters. DH5α transformant cells were selected on LB/Gen plates. The PBBR1MCS5PrpoDconsRFP (pBB*P*_*Eco*_) plasmid was digested with *Nhe*I to remove the DNA spacer and –10 box of the RpoD consensus promoter sequence, purified from agarose gel, and re-ligated. In this manner, pBBR1MCS5PlessRFP (pBB*P*_*less*_) was assembled. Once the pBBR1MCS5*P*_*n*_RFP (pBB*P*_*n*_) set was verified by sequencing, new external oligonucleotides were used to amplify the *P*_*n*_RFP fragment. *Bam*HI and *Sph*I restriction enzymes were used for pUC19*P*_*n*_RFP (pUC*P*_*n*_) construct and DH5α transformants were selected on LB/Amp plates at 37°C. All DNA amplification reactions were carried out with Platinum *Taq* High Fidelity DNA polymerase. We used the same DNA spacer sequence (located between –35 and –10 promoter boxes) for both consensuses (corresponding to promoter Bba_J23119 of the iGem repository).

### Bacterial growth curves measurements

*E.coli rpoD285* was transformed with pRK415sigma library and growth curves were recorded in Synergy 2 Microplate Reader (Biotek). Synergy 2 was set to record optical density at a wavelength of 600 nm (OD_600*nm*_) every 30 min within 24 h. Experiments were performed with five randomly selected colonies from each library member. We carried out four technical repetitions for each colony, giving a total of 20 per library member. For each experiment, bacterial colonies were picked from solid plates and inoculated in its corresponding microplate well (pre-culture at 30°C), which was then replicated into a fresh new one (30°C) and finally the preceding microplate was replicated and grown at 42°C. All technical repetition steps consisted of 24 h continuous growth with continuous fast shaking. For experiments using *E. coli* strain CAG1 (*E. coli rpoD800*) we randomly selected five colonies and performed two technical repetitions. In this way, we obtained 10 repetitions for each tested construction $$on *E. coli rpoD800*. Microplate (Nunclon, Thermo Scientific, model 167008) wells were filled with 200 μl of LB/Tet/0.5 mM IPTG liquid medium. Plate replication was accomplished using a 96-pin microplate replicator (Boekel, mod. 140500).

### Colony forming unit determination

Growth was also determined by counting colony forming units (CFU). Two colonies were randomly selected from the five biological replicates for each pRK415sigma library member screened previously. Two time points of the kinetic growth were sampled, 0 and 24 h, respectively. Microplate cultures were grown in the same conditions as that of growth curve experiments (200 μl of LB/Tet/0.5 mM IPTG on each well, 24 h kinetic, continuous fast shaking, permissive and restrictive temperatures, Biotek Reader). A pre-culture (30°C) was used for microplate replication in order to homogenize the starting OD_600nm_ of the sampled cultures. After replication, the microplate was grown at 42°C for 24 h. Samples were drawn from the later plate at each time point as follows: 10 μl from each well were added to independent eppendorf tubes containing 90 μl of 10 mM Magnesium Sulfate/0.01% Tween 40 solution. Starting samples were mixed roughly by vortexing, serially diluted and each construct dilution was split by dropping it into two independent LB/Tet/0.5 mM IPTG plates. One plate was left at permissive and the other at restrictive temperature, severally. In this manner, each biological replicate had two plates. When plates reached 24 h of growth, visible bacterial colonies were counted in droplets with manageable numbers. CFUs were calculated using the following formula: number of bacterial colonies counted on solid plate divided by the product of the droplet volume (ml) per total dilution of tube. A 96-pin microplate replicator was used for the replication step.

### Fluorescence measurements

For fluorescence measurements of RFP activity, three different colonies per *P*_*n*_RFP construct were randomly picked and the Biotek reader filters were set as follows: Excitation 530/25 nm, Emission 590/35 nm and gain 40. OD measurements were done as in the *E. coli rpo285* complementation experiments. *Rhizobium etli* OD measurements were performed at 620 nm every 2 h during 48 h at 30°C and fast shaking in liquid PY media. Finally, 96-well microplate for fluorescence readings (black walls, clear bottom and cover. Mod. 3904) were acquire from Corning Incorporated.

### DNA isolation and manipulation

Total deoxyribonucleic acid from *E. coli* DH5α and *R. etli* CFN42 was purified (Sambrook and Russell, 2001) and used as template for all wild type sequence amplification reactions. Plasmid purification was performed using High Pure Plasmid Isolation Kit (Roche) from overnight cultures. DNA amplification products were first separated by 1.2% agarose gel (w/v) electrophoresis (100 V, 60–75 min, 1X Tris-base/Acetic acid/EDTA buffer) and then isolated using High Pure PCR Product Purification Kit (Roche). DNA restriction enzyme digestions were done at two temperatures: 25°C (*ApaI* and *SmaI*) and 37°C (*Afl*II, *Bam*HI, *Kpn*I, *Nhe*I, *Spe*I, *Sph*I, and *Xba*I). DNA ligation was carried out using T4 DNA ligase at 16°C. DNA restriction and ligation reactions were left overnight. Restriction enzymes and DNA ligase were obtained from NEB. DNA amplification reactions were accomplished either with Platinum *Taq* High Fidelity or Phusion High Fidelity DNA polymerases. The first one yields a mixture of blunt-ended and 3′-A overhang products while the second only produces blunt-ended DNA fragments.

### DNA sequencing

The pRK415sigma library was sequenced with Sanger technology by Macrogen Inc. Each construct was sequenced in both forward and reverse strands. The pRK415*sigAmutant* set was sequenced by GenScript. The *P*_*n*_RFP library was sequenced at the Unidad de Sintesis y Secuenciacion de ADN, Instituto de Biotecnologia-UNAM.

### Data handling and statistical analysis

For data standardization, the ratio of measurements obtained at 42°C vs. those obtained at 30°C was calculated for each genetic construct, data type and time point.

Perl *ad hoc* scripts were used to format raw output files from the Biotek microplate reader. Sequence alignments were performed using MUSCLE (Edgar, 2004). Post-script and jpg files of the alignments were created with SeaView (Galtier et al., 1996) and Apache OpenOffice, respectively. Growth curve graphs and statistical analyses were done using suitable R software packages (ggplot2, statmod, stats; R Development Core Team, 2008, RRID:SCRRRID:SCR_001905). We used R package grofit for mathematically model growth curves (Kahm et al., 2010). Permutation tests were implemented with the R package compareGrowthCurves (Elso et al., 2004; Baldwin et al., 2007) using growth curves data. For hierarchical clustering, we chose Minkowski distances to determine groups according to median and median absolute deviation of standardized integral values. We performed Shapiro-Wilk, Anderson-Darling and Jarque-Bera tests to analyze normality. Wilcoxon U-tests were accomplished to determine groups of similar behaviors among constructs using standardized integral values (42/30°C) for *E. coli rpoD285*. Kendall rank correlation tests were carried out to assess correlation between: (1) OD and CFU standardized values, (2) integral parameters obtained from standardized RFP values (RFP/OD) and (3) integral parameters from *E*. *coli rpoD800* and DH5α. Due to extreme variation during the first 15 measurements of RFP activity, statistical analyses were done excluding these data points. The extremely high values of RFP activity could have arisen because cultures started from an overnight pre-culture reached stationary phase. Cells in this phase are expected to over-express the RFP. All Sanger sequence reads were handled using trace tuner for base-calling (Paracel/Celera, 2006), Lucy for sequence quality/trimming (Chou and Holmes, 2001) and MIRA (Chevreux et al., 1999, RRID:SCRRRID:SCR_010731) for assembling contigs. BLAST was used to obtain identity percentages (Altschul et al., 1990, RRID:SCRRRID:SCR_004870). The Psipred web server was employed for secondary structure prediction (Buchan et al., 2013). All programs, except for psipred, were run locally.

### Protein purification

*E. coli rpoD285* was used for this experiment. Three independent colonies of each pRK415sigma construct (excluding *sigA* mutants) were grown overnight at 30°C. New flasks containing 100 ml fresh media were inoculated with the previous cultures adjusting OD_600nm_ to 0.03 and left at 42°C. The OD_600nm_ of these cultures was periodically monitored and samples were extracted when they had reached 0.6–0.8. We adjusted the OD_600nm_ of each culture to obtain 40 ml at an OD_600nm_ of 0.6. From this point on, samples were kept on ice. The vector and constructs pRK*ch02, ch03, ch07, ch10*, and *ch11* reported an OD_600nm_ that ranges from 0.04 to 0.06 after 72 h at 42°C. These library members were excluded from this experiment. Cultures were washed with 1X PBS, re-suspended in ice-cold milliQ H_2_O supplemented with Protease Inhibitor Cocktail (Sigma-Aldrich) and sonicated three times (25 s, 13 Microns). Then, 5 ml of absolute acetone was added, samples were frozen overnight at –80°C and centrifuged. Pellets were re-suspended in 5 ml of Extraction Buffer (0.7 M Sucrose, 0.5 M Tris-base, 0.1 M KCl, 30 mM HCl, 50 mM EDTA, 2% β-Mercaptoethanol and PVPP), mixed with 6 ml of phenol and centrifuged. The aqueous phase was recovered, suspended in 15 ml of ammonium acetate, frozen overnight at –80°C and centrifuged. Samples were washed twice using 5 ml of 80% acetone. Samples were finally suspended in Solubilization Buffer (7 M Urea, 2 M Thiourea, 4% CHAPS, 2 mM TBP, 2% Ampholine, 600 mM DTT). Protein was quantified by Bradford Assay, using Bovine Serum Albumin as standard. All *E*. *coli rpoD*285 cultures were grown aerobically (220 rpm agitation) in liquid LB/Tet/0.5 mM IPTG media. All the centrifugation steps were done at 4°C, 7000 rpm for 20 min.

### Western blot

Protein samples (15 μl adjusted to 0.2 mg ml^−1^) were loaded onto 4–20% polyacrylamide gradient gels, PAG (BioRad, cat. 456-1095) and electrophored in 1X Tris-Gly-SDS buffer at 110 V for 2 h. Proteins were then electro-transferred from gels to nitrocellulose membranes (BioRad, cat. 162-0097) using a semi-dry chamber (400 mA, 1 h). After transfer, membranes were blocked overnight at 4°C in 5% non-fat dry milk TBST (10 mM Tris-HCl pH 8.0, 150 mM NaCl, 0.1% Tween 20), washed three times and incubated with primary antibody for 2 h at room temperature. Five washing steps were followed by incubation of membranes with secondary antibody for 1 h at room temperature. After another four washes, membranes were incubated in Carbazole Solution (27.2% Carbazole, 72.6% Acetate buffer, 0.2% H_2_O_2_). Primary antibody (mouse monoclonal IgG_2*b*_, code: 2G10, sc-56768, Creative Biomart Cat# CABT-36751ME, RRID:ABRRID:AB_11443551) targets primary sigma factors from a wide range of bacteria (Batut et al., 1991; Severinova et al., 1996; Breyer et al., 1997; Bowman and Kranz, 1998). Secondary antibody (goat anti-mouse IgG-HRP, Santa Cruz Biotechnology Cat# sc-2005, RRID:ABRRID:AB_631736) targets the first one and has the horseradish peroxidase conjugated. Primary and secondary antibodies were diluted 1:10,000 and 1:20,000, respectively. All membranes were arranged as follows: first row, protein ladder; second row, commercial *E*. *coli* Eσ^D^ (Epicentre); third row, *R. etli* CFN42 total protein sample and in the rest of the row, the remaining samples in alphabetic order. Washing steps were done at room temperature with TBST for 10 min each. Western blotting were done using gentle agitation (BenchRocker 2D, CORE Life Sciences). All antibodies were supplied by Santa Cruz Biotechnology Inc. PAGE-Ruler pre-stained protein ladder (10-180 kDa, Fermentas) and Loading Buffer (1.5 mM Tris, 10% SDS, β-Mercaptoethanol, Glycerol, Bromophenol blue, H_2_O) were used in all PAG. Electro-transfer: Anode-I Buffer (0.3 M Tris-HCl pH 10.4,10% methanol), Anode-II Buffer (25 mM Tris-HCl pH 10.4, 10% methanol) and Cathode Buffer (25 mM Tris-HCl pH 9.4, 40 mM Glycine, 10% methanol). Membranes were photographed using a Sony digital camera.

### Phylogenetic analysis

We selected a total of 74 sigma protein sequences belonging to 54 α-proteobacteria and 20 Enterobacteria species for the phylogenetic analysis. The protein sequences were aligned with ClustalW2 (Thompson et al., 2002, RRID:SCR_002909) and the evolutionary model (WAG) that best fit the data was obtained with ProtTest (version 2.4; Abascal et al., 2005). The phylogenetic reconstruction was made with PhyML software (version 3.0; Guindon and Gascuel, 2003).

### Sigma factor RpoD region σ4 crystal model

We selected and downloaded the PDB file 4YLN (Zuo and Steitz, 2015) from the PDB database (PDB, RRID:SCR_012820). This file contains the crystallographic structures of *E. coli* transcription initiation complexes comprising a complete transcription bubble. We obtained the chain F that belongs to RpoD and selected its region σ4 (amino acids 540 to 613). The structure of RpoD region σ4 and the promoter's -35 box DNA were display using the Swiss-PdbViewer software (version 4.1; Guex and Peitsch, 1997).

## Results

### *R. etli* SigA is laxer in promoter recognition than *E. coli* RpoD

**Figure 2:**
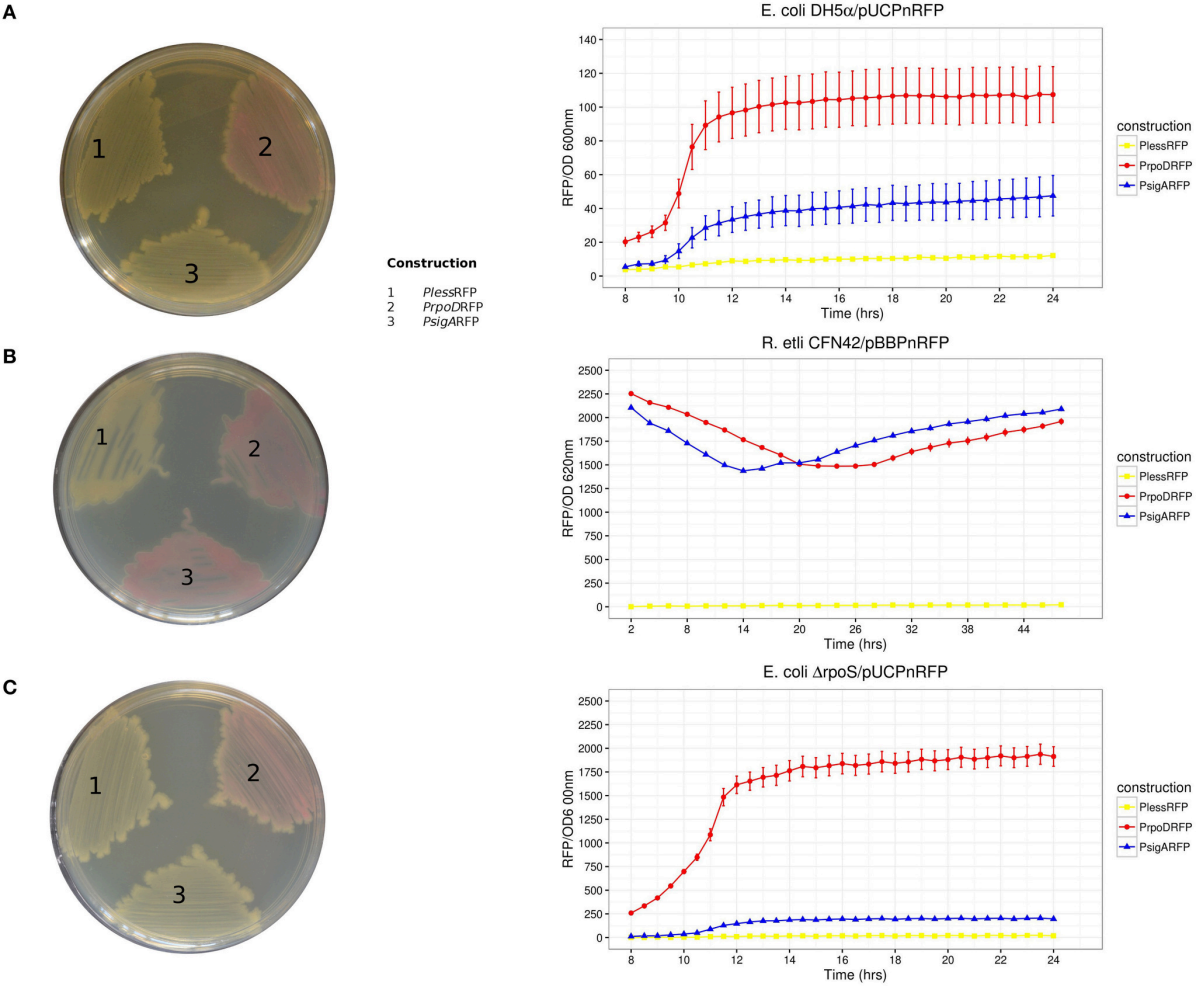
**RFP activities recorded as a kinetic curve (RFP/OD) and on solid culture plates**. Host strains: **(A)** *E. coli* DH5α, **(B)** *R. etli* CFN42, and **(C)** *E. coli* Δ*rpoS*. Solid culture plate's organization of transcriptional fusions: (1) *P*_*less*_RFP, (2) *P*_*Eco*_RFP, and (3) *P*_*Ret*_RFP. Reporter plasmids used were pUC19*Pn*RFP for both *E. coli* strains and pBBR1MCS5*Pn*RFP for *R. etli*. Three repetitions were done for each construction. Error bars denote SEM.

As described in Ramírez-Romero et al. (2006), the functional comparison between *E. coli* RpoD and *R. etli* SigA revealed that RpoD is stricter for promoter recognition, which is reflected in a robust consensus sequence (Table 3). The opposite case is observed among α-proteobacteria, where lax primary sigma factors allow a larger variation in its promoter structure (Karls et al., 1993; Malakooti et al., 1995; Cullen et al., 1997; MacLellan et al., 2006; Ramírez-Romero et al., 2006). This observation suggests that *E. coli* RpoD is unable to recognize *R. etli* SigA-dependent promoters or recognizes them less efficiently. In order to test this possibility, the red fluorescent protein (RFP) gene was put under the control of three different promoters: RpoD consensus sequence (*P*_*Eco*_), SigA consensus sequence (*P*_*Ret*_), and an RpoD consensus that lacks the spacer and –10 box (*P*_*less*_). Each of these constructs was cloned into pBBR1MCS5 (pBB*P*_*n*_) and pUC19 (pUC*P*_*n*_). The constructs pBB*P*_*Eco*_ and pBB*P*_*Ret*_ conferred a red color to *R*. *etli* CFN42 under previously described growth conditions, while the negative control pBB*P*_*less*_ remained white (Figure 2). These data showed that SigA is able to use the RpoD consensus promoter sequence to sustain expression of the reporter gene under the tested growth conditions.

**Table 3:** **Primary sigma promoter consensus sequences**.

**Species**	**–35 box**								**spacer**	**–10 box**								**References**
*E.coli*		T	T	G	A	C	A		15–19			T	A	T	A	A	T	Hawley and McClure, 1983
*R. etli*	C	T	T	G	A	C			16–23			T	A	T	N	N	T	Ramírez-Romero et al., 2006
*S. meliloti*	C	T	T	G	A	C			~17		c	T	A	T	a	t		MacLellan et al., 2006
*R. capsulatus*		T	T	G	A	C						AT-rich						Cullen et al., 1997
*C. crescentus*		T	T	G	A	C	G	S	10–14	G	C	T	A	N	A	W	C	Malakooti et al., 1995

*N, any nucleotide; S, C or G; W, A or T*.

At the same time, pUC*P*_*n*_ constructs were introduced into *E*. *coli* DH5α. Only pUC*P*_*Eco*_ displayed red colored colonies. Both pUC*P*_*Ret*_ and pUC*P*_*less*_ exhibited only white colored colonies (Figure 2). In order to exclude the participation of RpoS, the alternative sigma factor that shows partial resemblance to the RpoD consensus promoter sequence (Peano et al., 2015), we repeated the pUC*P*_*n*_ experiments using an *E. coli* Δ*rpoS* strain (*E. coli* BW28465. Zhou et al., 2003). RFP activities in *E. coli* Δ*rpoS* strain revealed good correlation to those observed on DH5α (Kendall τ = 0.72, *Pvalue* = 0.0091; Figure 2).

Taken together, these results confirm previous observations and provide new information supporting the following: (1) *E. coli* RpoD is unable to sustain gene expression under the control of the *R. etli* SigA consensus promoter sequence, (2) SigA is a primary sigma factor with a lax promoter recognition pattern, and (3) alternative sigma factors of *E*. *coli* recognize neither the RpoD nor SigA consensus promoter sequences.

### *R. etli* SigA gene complements the heat-sensitive phenotype of an *E. coli RpoD* mutant

**Figure 3:**
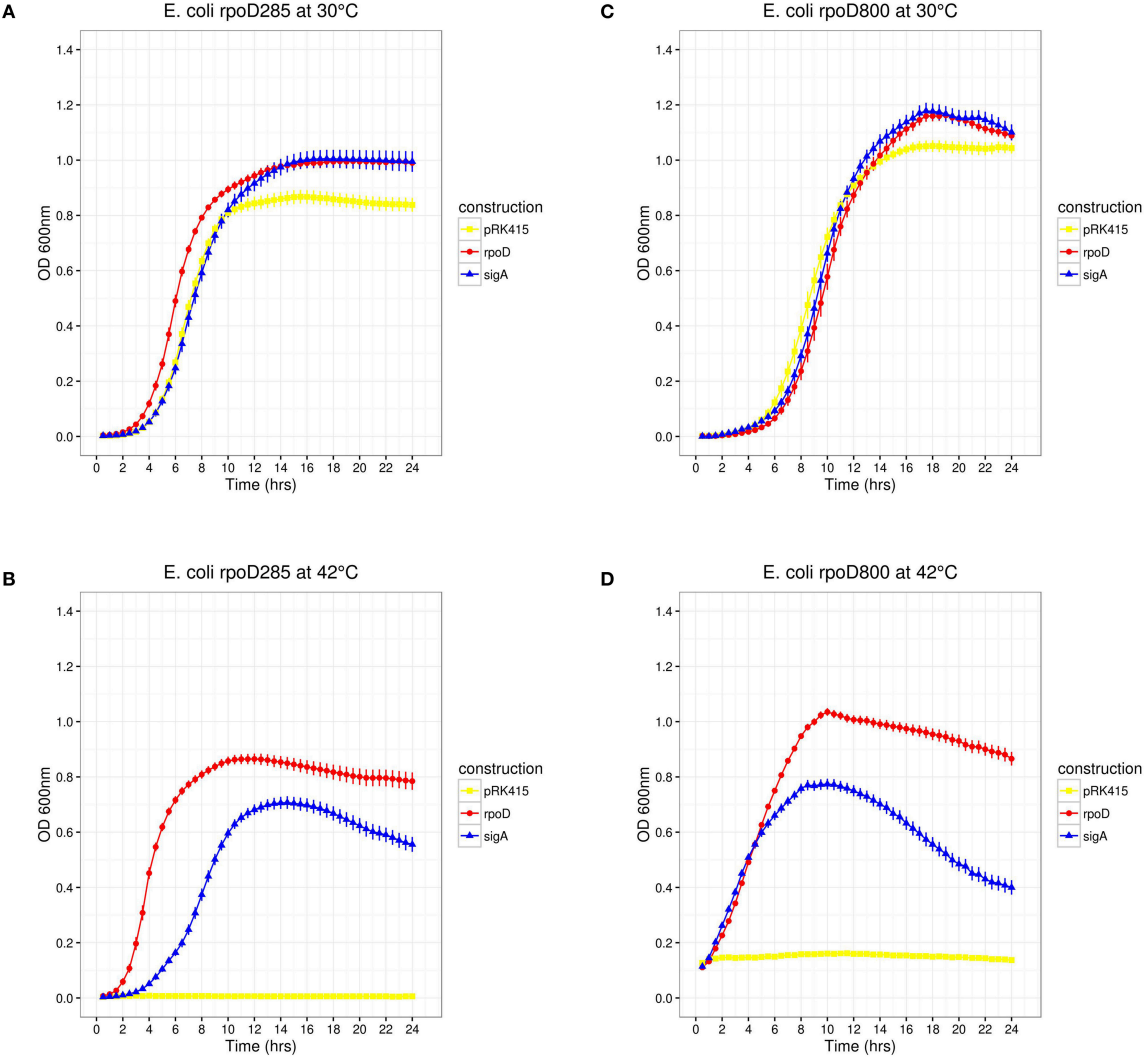
*****E. coli rpoD***285 and ***rpoD***800 complementation experiments**. pRK415, yellow squares; pRK*rpoD*, red circles; and pRK*sigA*, blue triangles. **(A)** *rpoD*285 at 30°C. **(B)** *rpoD*285 at 42°C. **(C)** *rpoD*800 at 30°C. **(D)** *rpoD*800 at 42°C. Number of repetitions: 20 for *E. coli rpoD*285 and 10 for *E. coli* rpoD800 experiments. Error bars denote SEM.

Previous results showed that transcriptional fusions containing 33 different *R. etli* SigA-dependent promoters are not recognized by *E. coli* RpoD. However, the *E. coli lactose* promoter (*P*_*lac*_) is recognized by SigA, suggesting that the latter is a laxer primary sigma factor [(Ramírez-Romero et al., 2006) and previous sections]. If this hypothesis were true, then SigA would be able to substitute RpoD *in vivo*. To test this, we used the *E*. *coli rpoD*285, which holds an RpoD thermo-sensitive allele. *rpoD*285 has a 42 bp in-frame deletion at its σNCR (Hu and Gross, 1983). This strain is unable to grow at 42°C (restrictive temperature) due to the unfolding of its primary sigma factor. At 30°C (permissive temperature) *E. coli rpoD*285 grows orderly (Harris et al., 1978; Hu and Gross, 1983). RpoD and SigA were separately cloned into pRK415 plasmid where they are expressed under the control of *P*_*lac*_. Constructs pRK415*rpoD* (pRK*rpoD*; positive control), pRK415*sigA* (pRK*sigA*), and the vector (pRK415, negative control) were independently transformed into *E. coli rpoD*285. All constructs grew at permissive temperature, presenting lag, exponential and stationary growth phases. Maximal stationary OD_600*nm*_ ranged between 0.8 and 1.0 (Figure 3A). At restrictive temperature, only the two primary sigma factor constructs sustained the growth of *E*.*coli rpoD*285, reaching maximal OD_600*nm*_ between 0.7 and 0.9 (Figure 3B). We also reproduced these experiments using another RpoD thermo-sensitive *E*. *coli* strain, CAG1 (*E. coli rpoD*800. Liebke et al., 1980). *E. coli rpoD*800 complementation results show moderate correlation to those from *rpoD*285 (Kendall τ = 0.669, *Pvalue* = 2.38 × 10^−7^). Maximal OD_600*nm*_ for *E. coli rpoD*800 experiments were: permissive temperature (1.0–1.2; Figure 3C) and restrictive temperature (0.75–1.0; Figure 3D). Neither *E*. *coli* strains *rpoD*285 nor *rpoD*800 were complemented by the vector at restrictive temperature. These results showed that *R. etli* SigA is able to complement the *E. coli* RpoD mutant phenotype.

### Construction of chimeric genes swapping RpoD and SigA regions

To identify regions involved in transcriptional laxity phenotype of SigA, we implemented a strategy based on the assembly of functional chimeric genes in *E. coli*. To this end, we constructed a library of 14 chimeras exchanging protein regions of RpoD and SigA (Figure 1). Each construct was designed in frame, maintaining intact the ORF. Chimeric genes were cloned into pRK415. To determine the functionality of these chimeras in *E. coli*, we chose *E*. *coli rpoD*285 as a host strain for complementation experiments.

## Identification of SigA regions conferring transcriptional laxity

### Substitution of SigA regions, one at a time

#### SigA σ1 and non-conserved regions are not involved in transcriptional laxity

**Figure 4:**
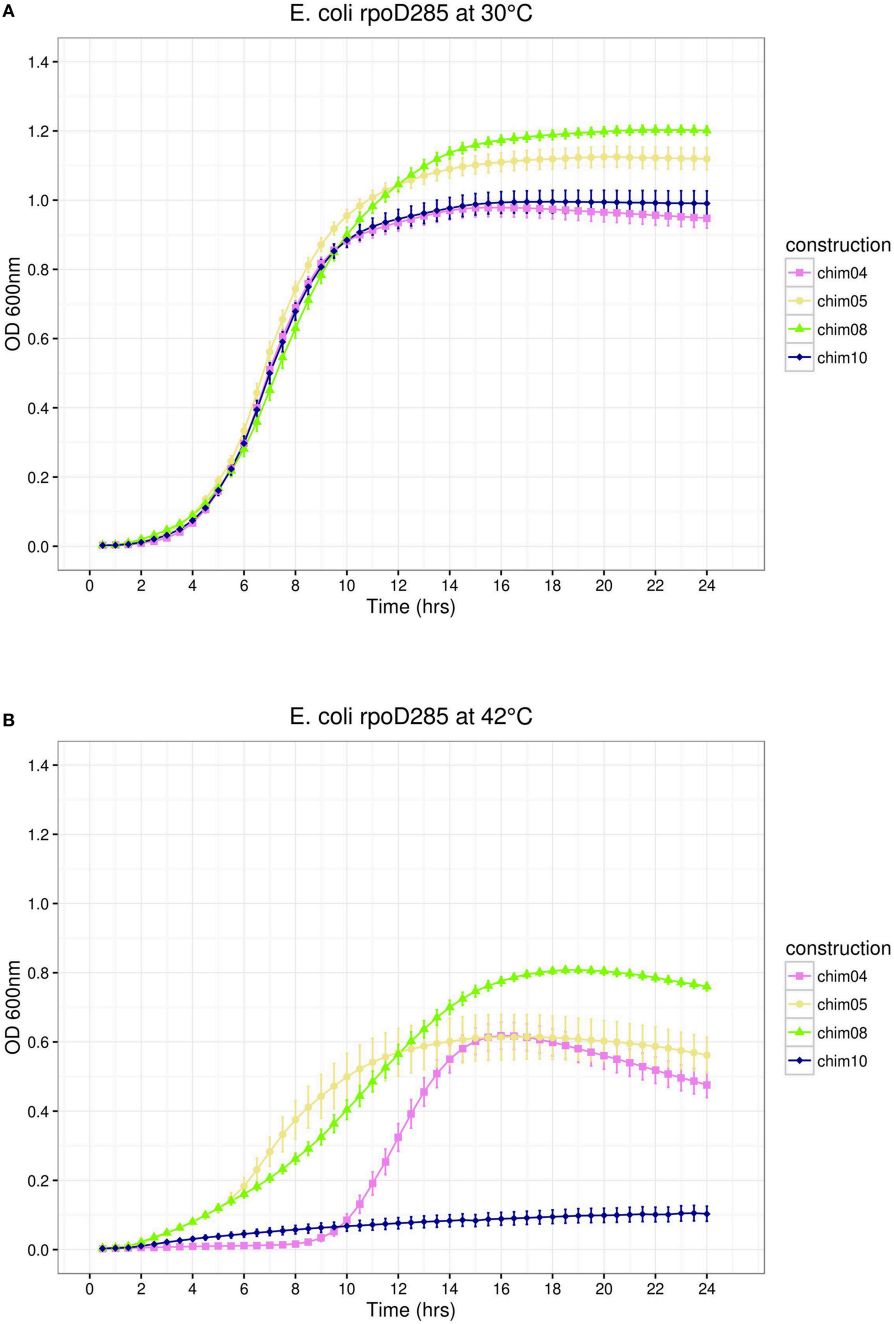
*****E. coli rpoD***285 complementation experiments**. pRK*ch04*, violet squares; pRK*ch05*, khaki circles; pRK*ch08*, chartreuse triangles; and pRK*ch10*, dark blue diamonds. **(A)** Growth temperature: 30°C. **(B)** Growth temperature: 42°C. Twenty repetitions were done for each construction. Error bars denote SEM.

The results described above suggest that SigA can recognize promoters associated with indispensable *E*. *coli* growth-related genes. The comparison of the predicted structure of SigA to known RpoD domains (Gruber and Gross, 2003) indicated that regions σ2.4 and σ4.2, responsible for promoter recognition, are identical and different by only three amino acids, respectively. SigA regions σ1 and σNCR comprise 72 extra residues, suggesting that differences located in these regions could be related to the SigA lax promoter recognition reported previously (Ramírez-Romero et al., 2006). If this were true, then the interchange of these SigA regions by their RpoD counterparts would change its promiscuous promoter recognition to a more stringent one. Chimeric constructs 04 and 05 (pRK*ch04* and pRK*ch05*) represent this design (Figure 1). *E*. *coli rpoD*285 growth complementation experiments showed the following: (1) At permissive temperature, these constructs displayed clear discernible growth phases. Maximal stationary OD_600*nm*_ ranged between 0.9 and 1.0 (Figure 4A).

(2) At restrictive temperature, all chimeras grew similarly to SigA, displaying growth phases. Maximal OD_600*nm*_ ranged from 0.8 to 0.9 (Figure 4B). According to these results, SigA regions σ1 and σNCR do not contain elements related to transcriptional laxity.

#### Neither SigA σ2 and σ3 regions participate in transcriptional laxity

Given that SigA and RpoD regions σ2 share 100% identity (100% coverage), it was discarded as a strong candidate for explaining transcriptional laxity. Construct pRK*ch04* supported this notion (see previous part). Furthermore, regions σNCR and σ2 are part of the same domain in RpoD (Gruber and Gross, 2003). For this reason, the design of chimeras 05 through 14 considered regions σNCR and σ2 as a unit.

Construct pRK*ch08* exchanged region σ3. It exhibited discernible growth phases at both temperatures. Maximal stationary OD_600nm_ reached 1.2 for permissive and 0.8 for restrictive temperatures (Figure 4). Because construct pRK*ch08* was able to complement *E. coli rpoD*285 thermo-sensitive phenotype, we concluded that SigA region σ3 do not participate in transcriptional laxity.

#### sigA σ4 region may participate in transcriptional laxity

The last chimeric construct (pRK*ch10*) swapped regions σ4. *E*. *coli rpoD*285 complementation experiments revealed the following: (1) At permissive temperature, this construct showed discernible growth phases, reaching maximal OD_600nm_ of 1.0 (Figure 4A) and (2) At restrictive temperature, this construct was unable to sustain cell growth (Figure 4B). This was the first observation suggesting that SigA region σ4 participates in transcriptional laxity.

To test possible interactions between regions regarding transcriptional laxity, we replaced two and three regions at a time in the remaining constructs (Figure 1). In this way, we can unveil the potential participation of more than one SigA region in transcriptional laxity.

### Substitution of SigA regions, two at a time

**Figure 5:**
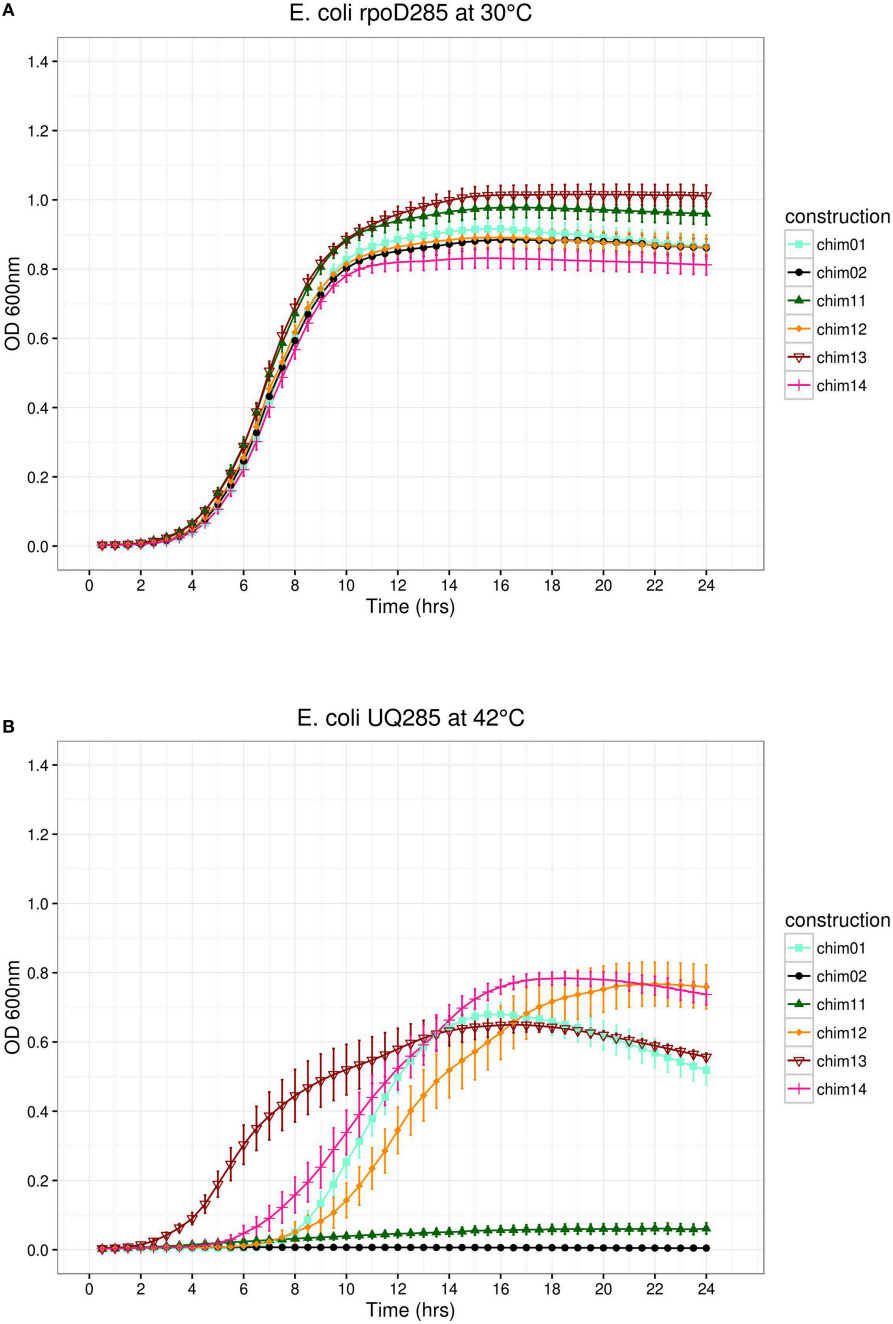
*****E. coli rpoD***285 complementation experiments**. pRK*ch01*, aquamarine squares; pRK*ch02*, black circles; pRK*ch11*, dark green triangles; pRK*ch12*, dark orange diamonds; pRK*ch13*, dark red open inverted triangles; and pRK*ch14*, deep pink plus symbols. **(A)** Growth temperature, 30°C. **(B)** Growth temperature, 42°C. Twenty repetitions were done for each construction. Error bars denote SEM.

This part of the pRK415sigma library was built exchanging every pair of regions according to a non-redundant combination design. In this way, six constructs were obtained. We replaced SigA regions in the following order: σ1/σ2 (pRK*ch01*), σ1/σ3 (pRK*ch13*), σ1/σ4 (pRK*ch11*), σ2/σ3 (pRK*ch12*), σ2/σ4 (pRK*ch14*), and σ3/σ4 (pRK*ch02*). All constructs behaved as expected at permissive temperature, displaying clear growth phases. Maximal stationary OD_600*nm*_ ranged between 0.8 and 1.1 (Figure 5A). At restrictive temperature, constructs pRK*ch02* and pRK*ch11* showed no growth. The other paired library members (pRK*ch01, ch12, ch13*, and *ch14*) displayed lag, exponential, and stationary growth phases with maximal OD_600nm_ ranging from 0.6 to 0.8 (Figure 5B). From previous results, we expected that constructions exchanging SigA region σ4 (pRK*ch02, ch11*, and *ch14*) would be unable to complement the *E. coli rpoD*285 phenotype. However, construction pRK*ch14* exhibited complementation capacity. We believe this ability is explained by the presence of both RpoD promoter's recognition regions (σ2 and σ4) and the functional compatibility between all its protein regions.

### Substitution of SigA regions, three at a time

**Figure 6:**
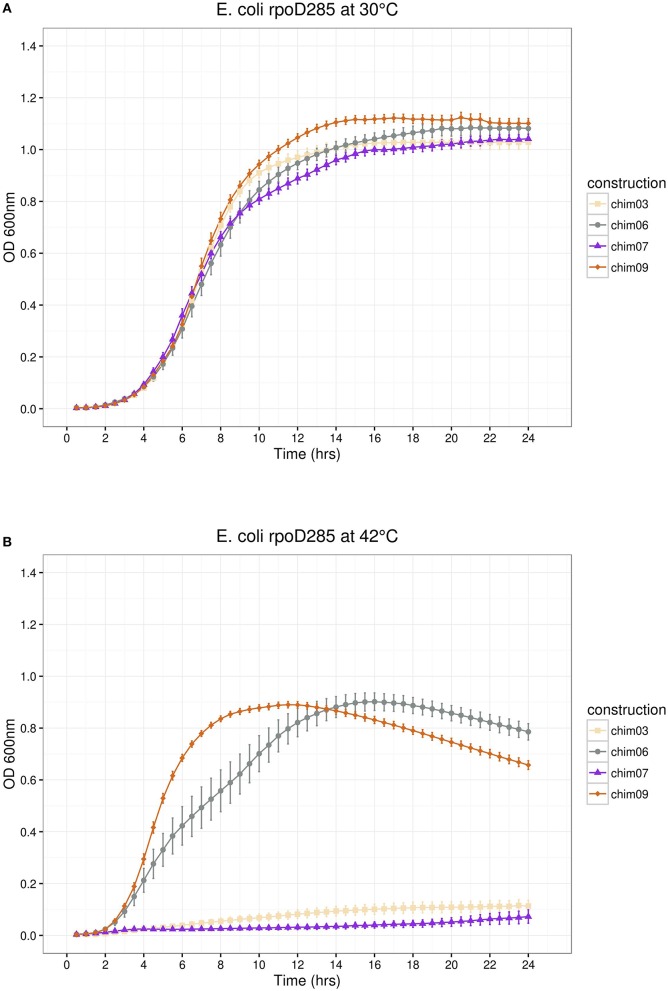
*****E. coli rpoD***285 complementation experiments**. pRK*ch03*, wheat squares; pRK*ch06*, azure 4 circles; pRK*ch07*, blue violet triangles and pRK*ch09*, chocolate diamonds. **(A)** Growth temperature, 30°C. **(B)** Growth temperature, 42°C. Twenty repetitions were done for each construction. Error bars denote SEM.

In the remaining part of the pRK415sigma library, we simultaneously interchanged three regions per construct. SigA replaced regions were: σ1/σ2/σ3 (pRK*ch09*), σ1/σ2/σ4 (pRK*ch07*), σ1/σ3/σ4 (pRK*ch03*), and σ2/σ3/σ4 (pRK*ch06*). At permissive temperature, all constructs showed growth, reaching maximal OD_600*nm*_ between 1.0 and 1.1 (Figure 6A). At restrictive temperature, constructs pRK*ch06* and *ch09* displayed growth (maximal OD_600nm_ of 0.9), but pRK*ch03* and *ch07* were unable to complement the *E*. *coli rpoD*285 thermo-sensitive phenotype (Figure 6B).

**Figure 7:**
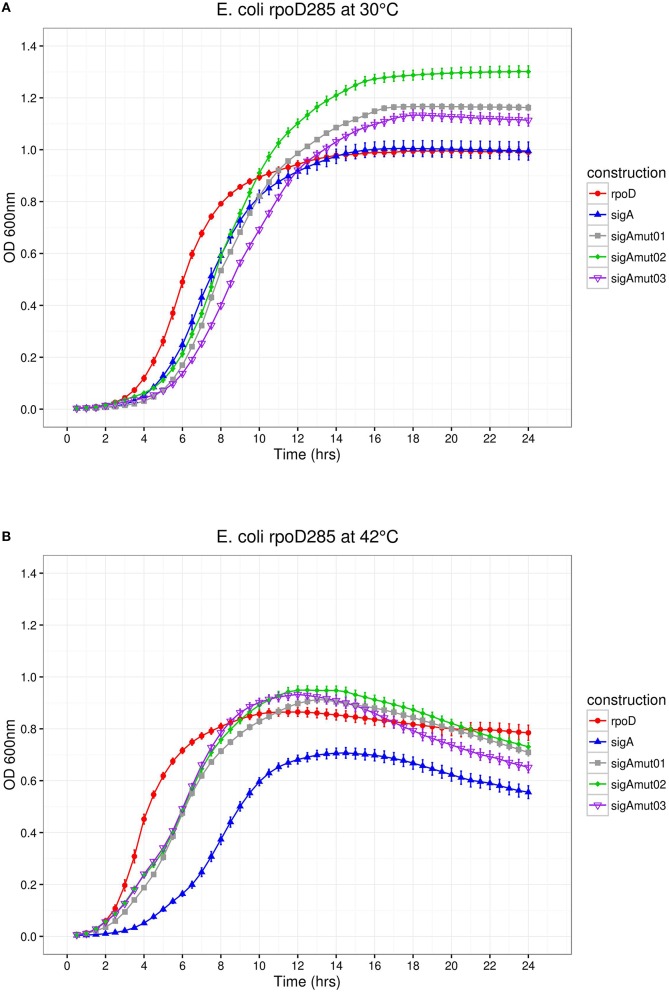
*****E. coli rpoD***285 complementation experiments**. PRK*rpoD*, red circles; pRK*sigA*, blue triangles; pRK*sigAm01*, gray squares; pRK*sigAm02*, green diamonds; and pRK*sigAm03*, purple open inverted triangles. **(A)** Growth temperature, 30°C. **(B)** Growth temperature, 42°C. Twenty repetitions were done for each construction. Error bars denote SEM.

For pRK415sigma library constructs that were able to sustain growth at 42°C, a general tendency of OD_600nm_ decay was observed after the culture had reached its maximum (Figures 3–7, sections B). This observation may be explained by the accumulation of metabolism by-products led by prolonged heat-stress. This may cause cell death and/or arrest in the tested conditions.

Constructs pRK*ch06, ch07*, and *ch14* hold the two RpoD regions known to be involved in promoter recognition (σ2 and σ4). Only pRK*ch07* was unable to complement the *E*. *coli rpoD*285 phenotype at restrictive temperature, although it displays 96% identity (100% coverage) to RpoD. The incapability of pRK*ch07* to complement *E. coli rpoD*285 phenotype suggests that this particular combination of domains renders the chimeric protein not functional, perhaps at RNAP core binding, transition from abortive RNA synthesis to transcription elongation (σ3) or misfolding of the entire protein. Given the previous results, the presence of chimeras that were unable to do it suggests RpoD-SigA regions incompatibility, maybe due to allosteric interactions.

### Chimeric and wild-type genes are translated in *E. coli rpoD*285

In order to establish if every pRK415 construct was translated in *E. coli rpoD*285, total protein was extracted from 14 library members grown at restrictive temperature. Constructs pRK*ch02, ch03, ch07, ch10*, and *ch11*, together with the empty vector, were discarded because of their inability to sustain growth at 42°C. Protein samples were collected, electrophoresed, electro-transferred to nitrocellulose membranes and incubated in primary antibody 2G10 (Creative Biomart Cat# CABT-36751ME, RRID:AB_11443551), which targets the amino acid region mapped from residues 470 to 486 on *E*. *coli* RpoD (inside region 3.1) and also cross-reacts to primary sigma factors of other bacterial species (Batut et al., 1991; Severinova et al., 1996; Breyer et al., 1997; Cullen et al., 1997; Bowman and Kranz, 1998). Western blot results showed a band corresponding to the molecular mass of RpoD (reference line two, *E*. *coli* Eσ^D^), suggesting the expression of all constructs that complemented growth of *E*. *coli rpoD*285 at restrictive temperature (Supplementary Figure 1).

### Colony forming units assay confirms the growth curve data

In pursuance of supporting growth curve data, we performed colony forming units (CFU) assay. CFU results showed general agreement with OD_600*nm*_ growth curve data (for statistical correlation tests please see Supplementary Information), i.e., only constructs that displayed growth on liquid media also did so on solid plates (Supplementary Figure 2).

### Growth curve analysis of the pRK415sigma library

To analyze the growth curves of pRK415sigma library, we mathematically modeled the observed data with gcFitModel function of R package grofit (Kahm et al., 2010). In this way, descriptive growth parameters as the lag phase length (λ), growth rate or maximum slope (μ), maximum cell growth (A), and the area under the curve (integral) were obtained. To review the goodness of the fit and statistical tests applied to these parameters please see Supplementary Information, Supplementary Figure 3; Supplementary Tables 1, 2.

The parameter integral was chosen to compare growth kinetics because this feature comprehends all the others. Clustering of standardized integral values (see Materials and Methods) allowed us to propose three groups of kinetic behaviors. No-growth group was integrated by the vector and chimera *ch02, ch03, ch07, ch10*, and *ch11*. Intermediate-growth group comprised *sigA* and chimera *ch01, ch04, ch05, ch08, ch12*, and *ch13*. High-growth group consisted of *rpoD*, chimera *ch06, ch09*, and *ch14*. For a more detailed description please see Supplementary Information and Supplementary Figure 4.

### SigA region σ4 aids transcriptional laxity

Growth curves and parameters data unveiled that each time SigA region σ4 appeared on a genetic construct, the carrier chimera was able to complement *E*. *coli rpoD*285 growth at restrictive temperature (Table 4). This feature was not observed with any other SigA region, i.e., regions σ1, σ2, and σ3 of *R*. *etli* primary sigma factor were present in chimeras that either sustain or impair growth at restrictive temperature. In order to test the sequence conservation of SigA region σ4 among the other α-proteobacteria that showed transcriptional laxity, we aligned the amino acid sequences of primary sigma factors from *R*. *etli, S*. *meliloti, R*. *capsulatus, R*. *sphaeroides, C*. *crescentus*, and *E*. *coli* using MUSCLE (Supplementary Figure 5). Sequence alignment revealed 16 mismatches out of 74 residues along region σ4 of these primary sigma factors. For this reason, we targeted these sites on SigA for mutational analysis.

**Table 4:** **Complementation analysis of pRK415sigma library**.

**Sigma region**	**Members surviving at 42**°**C**	
	**Yes**	**No**
SigA σ1	chim04,06,08,12,14	chim02,10
SigA σ2	chim05,08,13	chim02,03,10,11
SigA σ3	chim01,04,05,14	chim07,10,11
SigA σ4	chim01,04,05,08,09,12,13	0
RpoD σ1	chim01,05,09,13	chim03,07,11
RpoD σ2	chim01,04,06,09,12,14	chim07
RpoD σ3	chim06,08,09,12,13	chim02,03
RpoD σ4	chim06,14	chim02,03,07,10,11
SigA σ1, σ2	chim08	chim02,10
SigA σ1, σ3	chim04,14	chim10
SigA σ1, σ4	chim04,08,12	0
SigA σ2, σ3	chim05	chim10,11
SigA σ2, σ4	chim05,08,13	0
SigA σ3, σ4	chim01,04,05	0
RpoD σ1, σ2	chim01,09	chim07
RpoD σ1, σ3	chim09,13	chim03
RpoD σ1, σ4	0	chim03,07,11
RpoD σ2, σ3	chim06,09,12	0
RpoD σ2, σ4	chim06,14	chim07
RpoD σ3, σ4	chim06	chim02,03
SigA σ1, σ2, σ3	0	chim10
SigA σ1, σ2, σ4	chim08	0
SigA σ1, σ3, σ4	chim04	0
SigA σ2, σ3, σ4	chim05	0
RpoD σ1, σ2, σ3	chim09	0
RpoD σ1, σ2, σ4	0	chim07
RpoD σ1, σ3, σ4	0	chim03
RpoD σ2, σ3, σ4	chim06	0

*Constructions which hold the specified sigma factor region (first column) are separated according to its ability to complement (second column) or impair (third column) E. coli UQ285 growth at 42°C*.

### SigA region σ4 mutants

We decided to substitute the 16 mismatched positions found along region σ4 by exchanging SigA residues with its RpoD correspondents. These sequence changes should benefit SigA complementation capacity on *E*. *coli*. Secondary structure prediction using Psipred mapped this change within four different α-helices and some on its associated coil/looped regions. For SigA mutant 01 (*sigAm01*), five sequence changes were introduced into the first α-helix of region σ4; SigA mutant 02 (*sigAm02*) inserted four within the second α-helix and finally, SigA mutant 03 (*sigAm03*) inserted seven along the helix-turn-helix (HTH) motif. The HTH motif is responsible of promoter's −35 box recognition. Once the amino acid sequence mutants were designed, we identified its corresponding codons and exchanged them to those of RpoD. *In silico* designed *sigA* mutant sequences and pRK415 plasmid DNA were sent to GenScript (NJ, USA) to be chemically synthesized and cloned into the expression vector.

*E*. *coli rpoD*285 growth curve complementation and CFU experiments were repeated using the SigA mutant library. At both temperatures, SigA mutants displayed clear lag, exponential, and stationary growth phases (Figure 7). Maximal stationary OD_600nm_ ranged between values of 1.1–1.3 for permissive and 0.81–0.86 for restrictive temperatures. All SigA mutants were able to sustain growth of *E*. *coli rpoD*285 at restrictive temperature and exhibited OD decay during stationary phase (after reaching maximal growth) as previously seen on other pRK415sigma constructs. Growth parameters and statistical analysis for *sigA* mutants were computed as formerly described. The three SigA mutants fell into the high-growth cluster where RpoD resides (Table 5 and Supplementary Figure 4). All mutants improved complementation capacity compared to wild-type SigA, showing that sequence changes in region σ4 confer functional adaptation to *E. coli* transcriptional requirements. This sequence changes may diminish mutant proteins competence to transcribe *R*. *etli* SigA-dependent promoters. To test if pRK415sigma library members were able to transcribe RFP from pUC*P*_*Ret*_, we co-transformed *E*. *coli* DH5α with these two plasmids. None of the resulting transformants exhibited red colored colonies. Even the positive control, pRK*sigA*-pUC*P*_*Ret*_, featured only white colonies (data not shown). These findings imply that interactions between *E*. *coli* RNAP core, *R*. *etli* primary sigma factors and SigA consensus promoter sequence may not be appropriate enough to sustain transcription in *E. coli*.

**Table 5:** **Grouping of pRK415 sigma library members**.

**No**.	**Member**	**Description**	**42°C survive**	**Mean integral value**			**BLASTP**		**Growth group**
				**30°C**	**42°C**	**42°/30°**	**Identity %**	**coverage %**	
1	rpoD	σ1RpoD-σNCRσ2RpoD-σ3RpoD-σ4RpoD	yes	17.38	16.46	0.947	100	100	high
2	sigAmut03	σ1SigA-σNCRσ2SigA-σ3SigA-σ4SigAm3	yes	16.54	15.40	0.931	49	98	high
3	sigAmut01	σ1SigA-σNCRσ2SigA-σ3SigA-σ4SigAm1	yes	17.67	15.32	0.867	48	98	high
4	chim06	σ1SigA-σNCRσ2RpoD-σ3RpoD-σ4RpoD	yes	17.31	14.94	0.863	88	98	high
5	chim09	σ1RpoD-σNCRσ2RpoD-σ3RpoD-σ4SigA	yes	18.53	15.73	0.849	98	99	high
6	sigAmut02	σ1SigA-σNCRσ2SigA-σ3SigA-σ4SigAm2	yes	19.78	16.08	0.813	48	98	high
7	chim14	σ1SigA-σNCRσ2RpoD-σ3SigA-σ4RpoD	yes	13.95	10.36	0.743	84	98	high
8	sigA	σ1SigA-σNCRσ2SigA-σ3SigA-σ4SigA	yes	16.24	10.87	0.669	48	98	medium
9	chim13	σ1RpoD-σNCRσ2SigA-σ3RpoD-σ4SigA	yes	16.93	10.68	0.631	63	99	medium
10	chim08	σ1SigA-σNCRσ2SigA-σ3RpoD-σ4SigA	yes	18.93	11.51	0.608	51	98	medium
11	chim12	σ1SigA-σNCRσ2RpoD-σ3RpoD-σ4SigA	yes	14.90	8.60	0.577	85	98	medium
12	chim05	σ1RpoD-σNCRσ2SigA-σ3SigA-σ4SigA	yes	18.62	10.55	0.567	59	99	medium
13	chim01	σ1RpoD-σNCRσ2RpoD-σ3SigA-σ4SigA	yes	15.02	8.43	0.561	94	99	medium
14	chim04	σ1SigA-σNCRσ2RpoD-σ3SigA-σ4SigA	yes	16.41	7.02	0.428	73	98	medium
15	chim10	σ1SigA-σNCRσ2SigA-σ3SigA-σ4RpoD	no	16.68	1.79	0.108	50	98	no
16	chim03	σ1RpoD-σNCRσ2SigA-σ3RpoD-σ4RpoD	no	17.26	1.79	0.104	72	100	no
17	chim11	σ1RpoD-σNCRσ2SigA-σ3SigA-σ4RpoD	no	16.4	1.00	0.061	61	100	no
18	chim07	σ1RpoD-σNCRσ2RpoD-σ3SigA-σ4RpoD	no	16.74	0.87	0.052	96	100	no
19	chim02	σ1SigA-σNCRσ2SigA-σ3RpoD-σ4RpoD	no	14.75	0.20	0.014	54	98	no
20	pRK415	empty vector	no	14.63	0.19	0.013	NA	NA	no

*Integral values represent the area under the curve for each library member. RpoD sequence was used as query in BLASTP searches to determine identity percentages. Growth group determined by clustering library members. NA, not applicable*.

## Discussion

*R. etli* primary sigma factor, SigA, is able to transcribe most of the previously tested *E. coli* RpoD-dependent promoters (Ramírez-Romero et al., 2006), although these proteins locate on clearly separated phylogenetic clusters (Supplementary Figure 6). The same behavior was observed between other α-proteobacteria vs. the enterobacterial model (Karls et al., 1993; Malakooti et al., 1995; Cullen et al., 1997; MacLellan et al., 2006; Ramírez-Romero et al., 2006). This capacity may be explained, at least in part, by adaptive demands derived from the vast environmental conditions that α-proteobacteria inhabit.

In this work, we showed that SigA can complement *E. coli rpoD*285 (*rpoD* thermo-sensitive strain) growth at restrictive temperature (42°C). We have called this the transcriptional laxity phenomenon. Moreover, *R*. *etli* transcription machinery is also able to transcribe the RFP reporter gene from RpoD consensus promoter sequence. These results imply that the following conditions are met in *E*. *coli rpoD*285 host: (1) SigA is transcribed and translated, (2) SigA folds in a functional manner, (3) SigA is able to interact with RNAP core, assembling functional hybrid holoenzymes, (4) the hybrid holoenzymes are capable of transcribing indispensable genes for survival and growth at 42°C and (5) wild-type *R*. *etli* holoenzyme can transcribe the *E*. *coli* RpoD-dependent consensus promoter.

To identify the protein region(s) responsible for transcriptional laxity, we made a chimeric gene library exchanging regions of SigA and RpoD. In this way, all 14 non-redundant combinations between the two wild type genes were obtained. Chimeric library constructs were tested in their ability to complement *E. coli rpoD*285 growth at 42°C and these data were mathematically modeled to generate descriptive parameters of the construct kinetics.

We found that whenever SigA region σ4 is present in a chimera, the construct is able to complement *in vivo* the thermo-sensitive misfolding of RpoD285. Sequence alignment between *E*. *coli* RpoD and lax primary sigma factors from α-proteobacteria (Karls et al., 1993; Malakooti et al., 1995; Cullen et al., 1997; MacLellan et al., 2006; Ramírez-Romero et al., 2006), manifested 16 mismatches along region σ4. Sequence identity at these positions is conserved among α-proteobacterial proteins only. SigA mutant library was designed according to sequence alignment and secondary structure prediction data (Liebke et al., 1980; Buchan et al., 2013), resulting in three different mutant proteins. Although sigA and its mutants exhibit the lowest sequence identity percentage to RpoD among library members, the introduced changes significantly improved phenotypic complementation ability of SigA mutants (Wilcoxon U-test *Pvalues*: 1.02 × 10^−10^, 1.06 × 10^−7^, and 2.9 × 10^−11^) on *E*. *coli rpoD*285. This result shows that amino acid sequence identity alone is not enough to predict transcriptional laxity of a primary sigma factor.

Residues at specific positions within *E*. *coli* RpoD region σ4 play crucial roles during transcription, like: interaction with promoter's –35 box (R554, R562, D570, Y571, L573, E574, E575, G577, T583, R584, E585, R586, R588, Q589, K593, and R596; Hu and Gross, 1988; Siegele et al., 1988; Dombroski et al., 1992; Kim et al., 1995; Caslake et al., 1997; Campbell et al., 2002; Dove et al., 2003), RNAP core binding (E555, R562, F563, I565, G577, and L598; Sharp et al., 1999) and folding of the HTH motif (residues 575–581 and 585–608; Gruber and Gross, 2003). Among the 16 sequence changes in regions σ4 between RpoD and SigA, only six fell into critical positions described above. The corresponding residues-positions for RpoD/SigA are: Y571/H643, K578/Q650, D581/S653, R599/K671, E605/R677, and V606/K678. One sequence change (Y571/H643) lies in a position previously known to interact with the –35 box (Caslake et al., 1997). The remaining five positions reside inside HTH motif, K578/Q650 and D581/S653 are located on the first helix while R599/K671, E605/R677, and V606/K678 are situated on the second helix.

**Figure 8:**
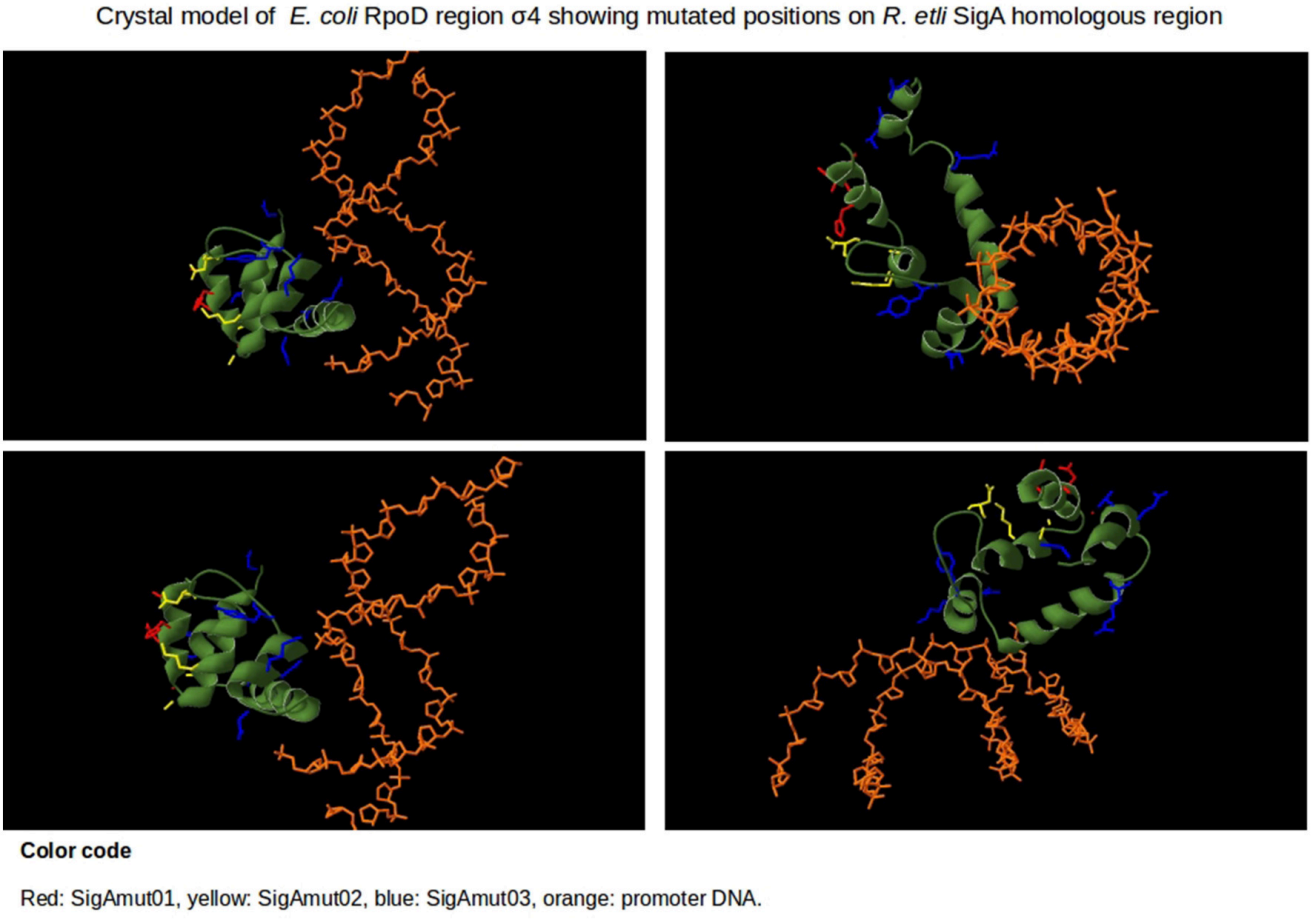
**Protein model structure of ***E. coli*** RpoD region σ4 (amino acids from position 540 to 613) and promoter's –35 box of 4YLN PDB file**. The 16 variable positions along region σ4 are shown in red, yellow, and blue. Position numbering is listed according to RpoD. SigAm01 (red): A542, A543, H545, D546, G550. SigAm02 (yellow): A553, A556, K557, D566. SigAm03 (blue): Y571, K578, D581, R599, E605, V606, D613.

The remaining variable positions occur on undefined function sites inside region σ4. The reported crystal structure of *E*. *coli* transcription initiation complex, PDB file 4YLN (Zuo and Steitz, 2015), revealed that these mismatched positions are not in close proximity to the promoter –35 box DNA (Figure 8). The first five variable residues (A542/E614, A543/T615, H545/T617, D546/R618, and G550/S622) are located on the first α-helix of region σ4 (represented by SigAm01). The next four (A553/P625, A556/E628, K557/R629, and D566/G638) reside on the second α-helix (SigAm02). Finally, the remaining seven mismatched positions (Y571/H643, K578/Q650, D581/S653, R599/K671, E605/R677, V606/K678, and D613/S685) occur inside the HTH motif (SigAm03). SigAm03 was the construct that best complements *E*. *coli rpoD*285 phenotype at restrictive temperature, followed by SigAm01. SigAm02 also significantly increased complementation capacity compared to the wild-type protein (Table 5). Taken together, these results imply that the first two α-helices are necessary for correct folding of region σ4 and are as essential as the HTH motif itself.

In this study, we show that region σ4 is involved in transcriptional laxity, at least among α-proteobacterial primary sigma factors. Moreover, sequence changes in this protein region alone can enhance its transcriptional capacity on the target host organism. This transcriptional improvement can be achieved by altering the first two α-helices of region σ4, although they do not appear to directly interact with promoter DNA. Comparison of primary sigma dependent promoter consensus sequences between *E*. *coli* and α-proteobacteria revealed that –35 boxes are strongly conserved while –10 boxes display higher sequence variation in the latter bacterial class (Table 3). These observations support the relevance of region σ4 in transcriptional laxity phenomenon among α-proteobacteria.

We propose that primary sigma factors of the α-proteobacteria class rely mostly on region 4 to achieve transcription. It is also the main target region for sequence changes that enhance functional fitness of the protein to its host organism. The first two α-helices of region σ4 may help in efficient folding and positioning of the HTH motif, so recognition of the –35 box could be accomplished. We also hypothesize that α-proteobacterial primary sigma factors depend predominantly on finding and binding to the –35 box of the promoter sequence during closed complex formation. Region σ4 and –35 box interaction anchors Eσ at the promoter long enough to start transcription bubble, lessening the need for a well conserved –10 box. In this way, α-proteobacteria manage to sustain transcription from a wide variety of promoter sequences, even from those of other bacterial classes of its phylum. Transcriptional laxity may have arisen during α-proteobacterial evolution to: (1) ensure expression of essential genes on vast environmental conditions, (2) exploit possible advantageous sequences obtained by horizontal gene transfer, (3) adapt and colonize the vast environments they inhabit today, and (4) counteract the effect of naturally occurring mutations at endogenous promoter sequences.

## Author contributions

OS, MR, and GD designed the study. OS performed the statistical analysis and the majority of the experiments. OS and AC purified the protein samples and carried out Western Blotting. LL built the phylogenetic trees and crystal model figures of region σ4. OS wrote the paper and prepared figures. MR, GD, and SE critically edited the manuscript.

## Funding

This work was supported by Consejo Nacional de Ciencia y Tecnología (CONACYT), México (project 154833), and Universidad Nacional Autónoma de México.

### Conflict of interest statement

The authors declare that the research was conducted in the absence of any commercial or financial relationships that could be construed as a potential conflict of interest.
