# Supplementary material for: Region 4 of Rhizobium etli Primary Sigma Factor (SigA) Confers Transcriptional Laxity in Escherichia coli
Source: Front Microbiol. 2016 Jul 13;7:1078. doi: 10.3389/fmicb.2016.01078 (PMC4943231; doi:10.3389/fmicb.2016.01078)
Supplement: Supplementary file 1 [file DataSheet2.pdf]

Supplementary Material
Region 4 of Rhizobium etli primary sigma factor (SigA) confers transcriptional laxity in Escherichia coli

Orlando Santillán*, Miguel Angel Ramírez-Romero*, Luis Lozano, Alberto Checa, Sergio Encarnación and Guillermo Dávila

*** Correspondence:** Orlando Santillán: osantillan@lcg.unam.mx
Miguel A. Ramírez: mramirez@oncomedicmexico.com

1 Supplementary Tables

Supplementary Table 1. N: number of fitted curves; SD: standard deviation; SEM: standard error of the mean.

Supplementary Table 1. Goodness of the fit									
No.	Construction	30 ºC				42 ºC			
		N	Residual sum of squares			N	Residual sum of squares		
			Mean	SD	SEM		Mean	SD	SEM
1	pRK415	20	0.006331	0.004061	0.000908	13	0.000051	0.000047	0.000013
2	rpoD	20	0.010426	0.005696	0.001274	20	0.081604	0.118154	0.026420
3	sigA	20	0.008611	0.008336	0.001864	20	0.103099	0.085543	0.019128
4	chim01	20	0.011582	0.009279	0.002075	20	0.104611	0.074134	0.016577
5	chim02	20	0.006548	0.004993	0.001116	9	0.000200	0.000524	0.000175
6	chim03	20	0.006500	0.003094	0.000692	19	0.000522	0.000678	0.000155
7	chim04	20	0.009232	0.004902	0.001096	20	0.056920	0.037550	0.008396
8	chim05	20	0.007556	0.003268	0.000731	19	0.049328	0.058701	0.013467
9	chim06	20	0.009415	0.008475	0.001895	20	0.094748	0.086242	0.019284
10	chim07	20	0.015392	0.008700	0.001945	18	0.001652	0.003960	0.000933
11	chim08	20	0.005027	0.001817	0.000406	20	0.017904	0.005996	0.001341
12	chim09	20	0.014440	0.014053	0.003142	20	0.192658	0.058816	0.013152
13	chim10	20	0.006806	0.004154	0.000929	18	0.001152	0.001389	0.000327
14	chim11	20	0.004848	0.002739	0.000613	19	0.000451	0.000562	0.000129
15	chim12	20	0.009273	0.006790	0.001518	20	0.014063	0.015370	0.003437
16	chim13	20	0.008809	0.004236	0.000947	20	0.091158	0.073389	0.016410
17	chim14	20	0.006782	0.005677	0.001269	20	0.040184	0.055403	0.012389
18	sigAmut01	20	0.004882	0.001297	0.000290	20	0.129836	0.069024	0.015434
19	sigAmut02	20	0.004666	0.001816	0.000406	20	0.166764	0.037405	0.008364
20	sigAmut03	20	0.005474	0.002086	0.000466	20	0.313577	0.128724	0.028784

Supplementary Table 2. S-W: Shapiro-Wilk test; A-D: Anderson-Darling test; J-B: Jarque-Bera test. *E. coli* BW28465 (Δ*rpoS*) RFP values were obtained by dividing RFP/OD measurements. This experiments were done at 37ºC. *E. coli* DH5α RFP values were obtained in the same way as those for strain BW28465. *E. coli* CAG1 (*rpoD*800). *E. coli* UQ285 (*rpoD*285).

Supplementary Table 2. Normality tests

Parameter	*Pvalues* for 30ºC data			*Pvalues* for 42ºC data		
	S-W	A-D	J-B	S-W	A-D	J-B
E. coli Δ*rpoS* RFP integral	0.002	0.001	0.47			
E. coli CAG1 *rpoD*800 integral				0.0002	2.7×10^{-5}	0.2
DH5α RFP integral	0.08	0.13	0.6			
*E. coli rpoD*285 growth rate (μ)	5.7×10^{-13}	2.2×10^{-16}	3.8×10^{-12}	2.2×10^{-16}	2.2×10^{-16}	1.8×10^{-8}
*E. coli rpoD*285 integral (I)	0.0004	0.0002	0.01	4.7×10^{-14}	2.2×10^{-16}	1.6×10^{-7}
*E. coli rpoD*285 lag phase length (λ)	0.62	0.48	0.91	1.4×10^{-9}	6.7×10^{-15}	1.9×10^{-9}
*E. coli rpoD*285 maximum cell growth (A)	1.5×10^{-6}	2.3×10^{-8}	0.0004	2.2×10^{-16}	2.2×10^{-16}	1.3×10^{-11}
*E. coli rpoD*285 OD_{600nm} at 0 hrs	0.02	0.02	0.25			
*E. coli rpoD*285 CFU at 0 hrs	7.2×10^{-6}	9.6×10^{-9}	0.006			
*E. coli rpoD*285 OD_{600nm} at 24 hrs	0.01	0.004	0.44			
*E. coli rpoD*285 CFU at 24 hrs	5.1×10^{-7}	1.1×10^{-10}	7.2×10^{-10}			
*E. coli rpoD*285 integral 42º/30ºC	4.7×10^{-15}	2.2×10^{-16}	4.4×10^{-8}			

Supplementary Table 3. Only pairs of constructions that failed to reject H_0 (*Pvalue* < 0.05/190) are present in the table. H_0: the two constructions come from the same population. Standardized integral values were obtained by dividing 42ºC/30ºC data.

Supplementary Table 03. Wilcoxon test for standardized integral values

Group 1	Group 2	*P value*
pRK415	chim02	0.0956
	chim03	0.0542
	chim07	0.0197
	chim10	0.0197
	chim11	0.4484
rpoD	chim06	0.0911
	chim09	0.0018
	sigAmut01	0.0014
	sigAmut03	0.4135
sigA	chim01	0.0051
	chim05	0.1661
	chim08	0.0181
	chim12	0.3408
	chim13	0.6588
	chim14	0.2110
chim01	chim04	0.0020
	chim05	0.5687
	chim08	0.1207
	chim12	0.3834
	chim13	0.1022
	chim14	0.0005
chim02	chim03	0.0186
	chim07	0.0075
	chim10	0.0019
	chim11	0.0607
chim03	chim07	0.2844
	chim10	0.4252
	chim11	0.1378
chim04	chim05	0.0069
	chim12	0.0051
chim05	chim08	0.8567
	chim12	0.8134
	chim13	0.3088
	chim14	0.0128
chim06	chim09	0.2012
	chim14	0.0181
	sigAmut01	0.4612
	sigAmut02	0.0263
	sigAmut03	0.2315
chim07	chim10	0.1610
	chim11	0.6415
chim08	chim12	0.7584
	chim13	0.1572
	chim14	0.0020
chim09	chim14	0.0167
	sigAmut01	0.1738
	sigAmut02	0.0559
	sigAmut03	0.0006
chim10	chim11	0.0120
chim12	chim13	0.6783
	chim14	0.0460
chim13	chim14	0.1207
chim14	sigAmut01	0.0095
	sigAmut02	0.1081
sigAmut01	sigAmut02	0.0004
	sigAmut03	0.0073

2 Supplementary Figures

Supplementary Figure Legends

Supplementary Figure 1. Western blot of *E. coli* UQ285 (*rpoD*285). Lane 1: Commercial Eσ^{70} from *E. coli* (Epicentre); lane 2: Total protein sample from *R. etli* CFN42 grown at 30ºC, lane 3: pRK415, lane 4: pRK*rpoD*, lane 5: pRK*sigA*, lane 6 pRK*ch01*; lane 7: pRK*ch02*, lane 8: pRK*ch03*, lane 9: pRK*ch04*, lane 10: pRK*ch05*, lane 11: pRK*ch06*, lane 12: pRK*ch07*, lane 13: pRK*ch08*, lane 14: pRK*ch09*, lane 15: pRK*ch10*, lane 16: pRK*ch11*, lane 17: pRK*ch12*, lane 18: pRK*ch13*, lane 19: pRK*ch14*, lane 20: pRK*rpoD*, lane 21: pRK*sigA*, lane 22: pRK*ch01*, lane 23: pRK*ch04*, lane 24: pRK*ch05*, lane 25: pRK*ch06*, lane 26: pRK*ch08*, lane 27: pRK*ch09*, lane 28: pRK*ch12*, lane 29: pRK*ch13* and lane 30: pRK*ch14*. Growth temperature of samples: 30ºC (lanes 3–19) and 42ºC (20–30). 15 µl [0.2 mg ml^{-1}] of each sample were loaded onto the indicated well. For *E. coli* Eσ^{70}, 1 µl of the holoenzyme was suspended in Solubilization Buffer to adjust final volume to 15 µl.

Supplementary Figure 2. Two biological replicates were selected to perform CFU count on solid plates. Two time points were sampled (0 and 24 hours) for each temperature (permissive or restrictive). Error bars denote SEM.

Supplementary Figure 3. Observed data: blue. Gompertz data: green. Logistic data: red. Gompertz and logistic equations used the parameters obtained by modelling the observed data. A) 30ºC data. B) 42ºC data.

Supplementary Figure 4. Minkowski distances were used to determine groups among pRK415sigma library members according to median and median absolute deviation of standardized integral values (42ºC/30ºC).

Supplementary Figure 5. Amino acid sequence of primary sigma factors from *C. crescentus*, *E. coli*, *R. capsulatus*, *R. etli*, *R. sphaeroides* and *S. meliloti* were aligned using MUSCLE.

Supplementary Figure 6. Phylogenetic tree reconstruction of some primary sigma factor proteins from Enterobacteria and α-proteobacteria.

Supplementary Information

Colony forming units assay confirms the growth curve data

Standardized OD and CFU data were used for statistical correlation tests (Methods). For the time point 0 hours, we found no correlation between OD and CFU data (Kendall τ = -0.1, *P-value* = 0.55). This might be explained by the low number of bacteria present at the beginning of the kinetic. At 24 hours, OD and CFU data displayed a moderate correlation (Kendall τ = 0.48, *P-value* = 0.0037). Given that all pRK415sigma library members exhibited bacterial colonies on plates at permissive temperature for both time points, we adduce that genetic constructs pRK*ch02*, *ch03*, *ch07*, *ch10*, and *ch11* together with the empty vector enter cell division arrest at 42ºC growth, i.e. these constructs remain viable at least during the first 24 hours.

Growth curve analysis of the pRK415sigma library

To analyze the growth curves of pRK415sigma library, we mathematically modelled the observed data with gcFitModel function of R package grofit (Kahm et al. 2010). In this way, descriptive growth parameters as the lag phase length (λ), growth rate or maximum slope (μ), maximum cell growth (A) and the area under the curve (integral) were obtained.

To assess the goodness of the fit, i.e., how well the models described the observed data, we calculated the Residual Sum of Squares (RSS) for the fitted observations (Supplementary Table 1). The mean RSS was 0.0081 (standard error of the mean, SEM = 0.00068) for the 30ºC fitted growth curves and 0.073 (SEM = 0.01826) for 42ºC counterparts. These RSS values showed that the observed data were successfully fitted by the mathematical models, making growth parameters reliable descriptors for these bacterial kinetics. For visualization purposes, we also plot the observed *versus* modelled data (Gompertz and Logistic equations) (Supplementary Figure 3).

Statistical tests to assess normality were applied to growth parameters obtained at permissive and restrictive temperatures. *P-values* were computed using Shapiro-Wilk, Anderson-Darling and Jarque-Bera tests (stats, nortest and tseries packages from R, respectively). At a significance level of $\alpha = 0.05$, all of the normality statistical tests indicated that none of the parameters have a Gaussian distribution, except for values of parameter λ at 30ºC (e.g. Shapiro-Wilk *P-value* = 0.617) (Supplementary Table 2). For this reason, non-parametric tests were selected to compare construct behaviors based on its descriptive parameters.

The parameter integral was chosen to compare growth kinetics because this feature comprehends all the other parameters. Integral values obtained from curves grown at 42ºC were standardized in respect to its corresponding permissive temperature results (integral 42 divided by integral 30). A standardized integral value of 1.0 implies that the two growth curves had the same parameter values at both temperatures tested. Wilcoxon test and hierarchical clustering (using Minkowski distances, Supplementary Figure 4) were performed over the standardized data set, allowing us to propose three groups of kinetic behaviors. No-growth group was integrated by pRK415 and chimera *ch02*, *ch03*, *ch07*, *ch10* and *ch11*. Intermediate-growth group comprised *sigA* and chimera *ch01*, *ch04*, *ch05*, *ch08*, *ch12* and *ch13*. High-growth group consisted of *rpoD*, chimera *ch06, ch09* and *ch14*. The only discrepancy between the groups proposed by the two methods was that in Minkowski clustering, *ch14* was located on the High-growth group while it could be assigned into both Intermediate and High-growth clusters by the Wilcoxon test. We also applied a permutation test over growth curves (R function compareGrowthCurves) and these results were in agreement with those from the Wilcoxon test (data not shown).
