# Supplementary material for: Region 4 of Rhizobium etli Primary Sigma Factor (SigA) Confers Transcriptional Laxity in Escherichia coli
Source: Front Microbiol. 2016 Jul 13;7:1078. doi: 10.3389/fmicb.2016.01078 (PMC4943231; doi:10.3389/fmicb.2016.01078)

Supplementary Figure 1

A

Western Blot of *E. coli* UQ285/pRK415sigma library. Repetition 1

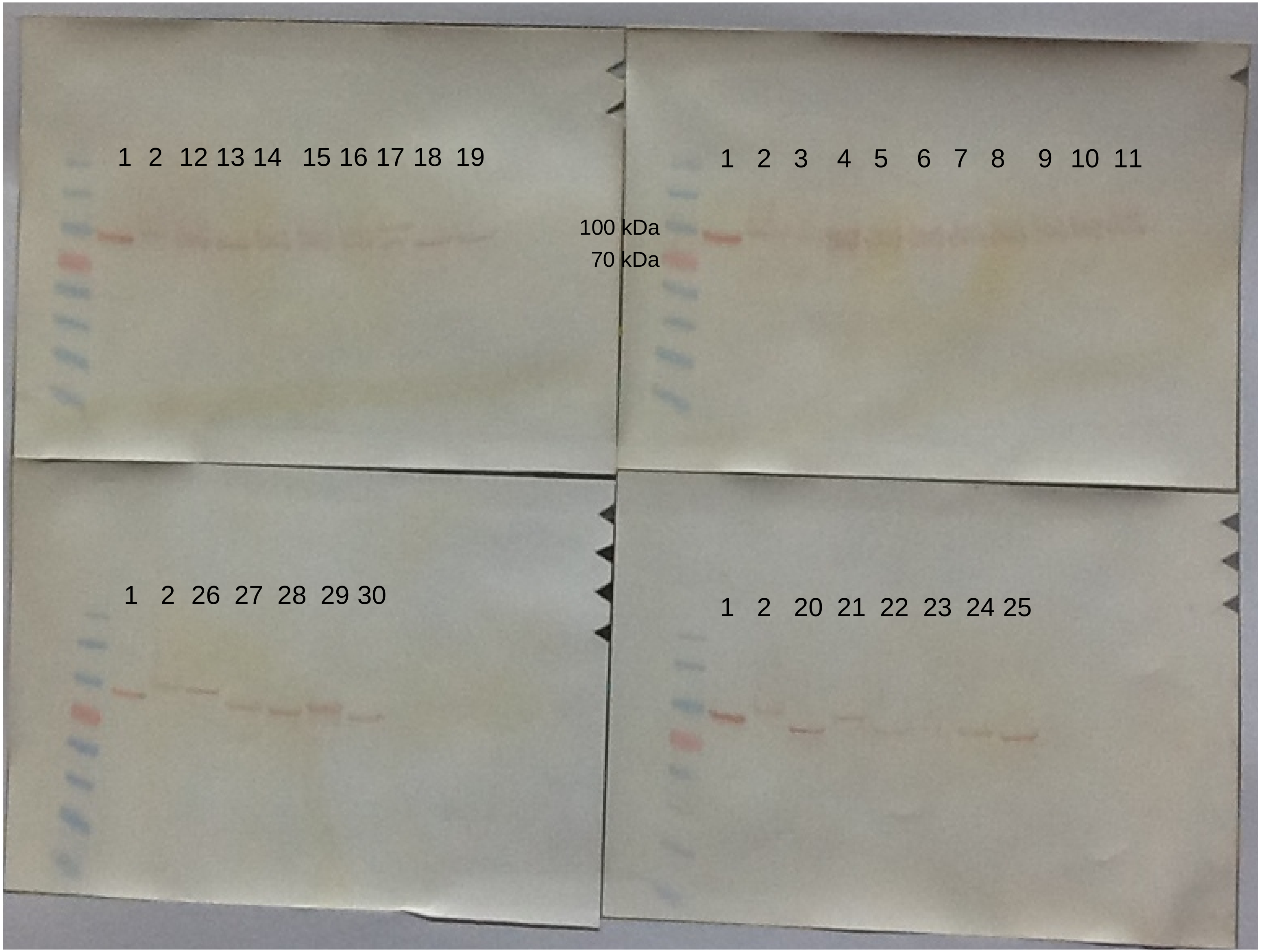

No.	Sample
1	Eσ70 *E. coli*
2	SigA *R. etli*
3	pRK415 30ºC
4	RpoD 30ºC
5	SigA 30ºC
6	Chim01 30ºC
7	Chim02 30ºC
8	Chim03 30ºC
9	Chim04 30ºC
10	Chim05 30ºC
11	Chim06 30ºC
12	Chim07 30ºC
13	Chim08 30ºC
14	Chim09 30ºC
15	Chim10 30ºC
16	Chim11 30ºC
17	Chim12 30ºC
18	Chim13 30ºC
19	Chim14 30ºC
20	RpoD 42ºC
21	SigA 42ºC
22	Chim01 42ºC
23	Chim04 42ºC
24	Chim05 42ºC
25	Chim06 42ºC
26	Chim08 42ºC
27	Chim09 42ºC
28	Chim12 42ºC
29	Chim13 42ºC
30	Chim14 42ºC

B

Western Blot of *E. coli* UQ285/pRK415sigma library. Repetition 2

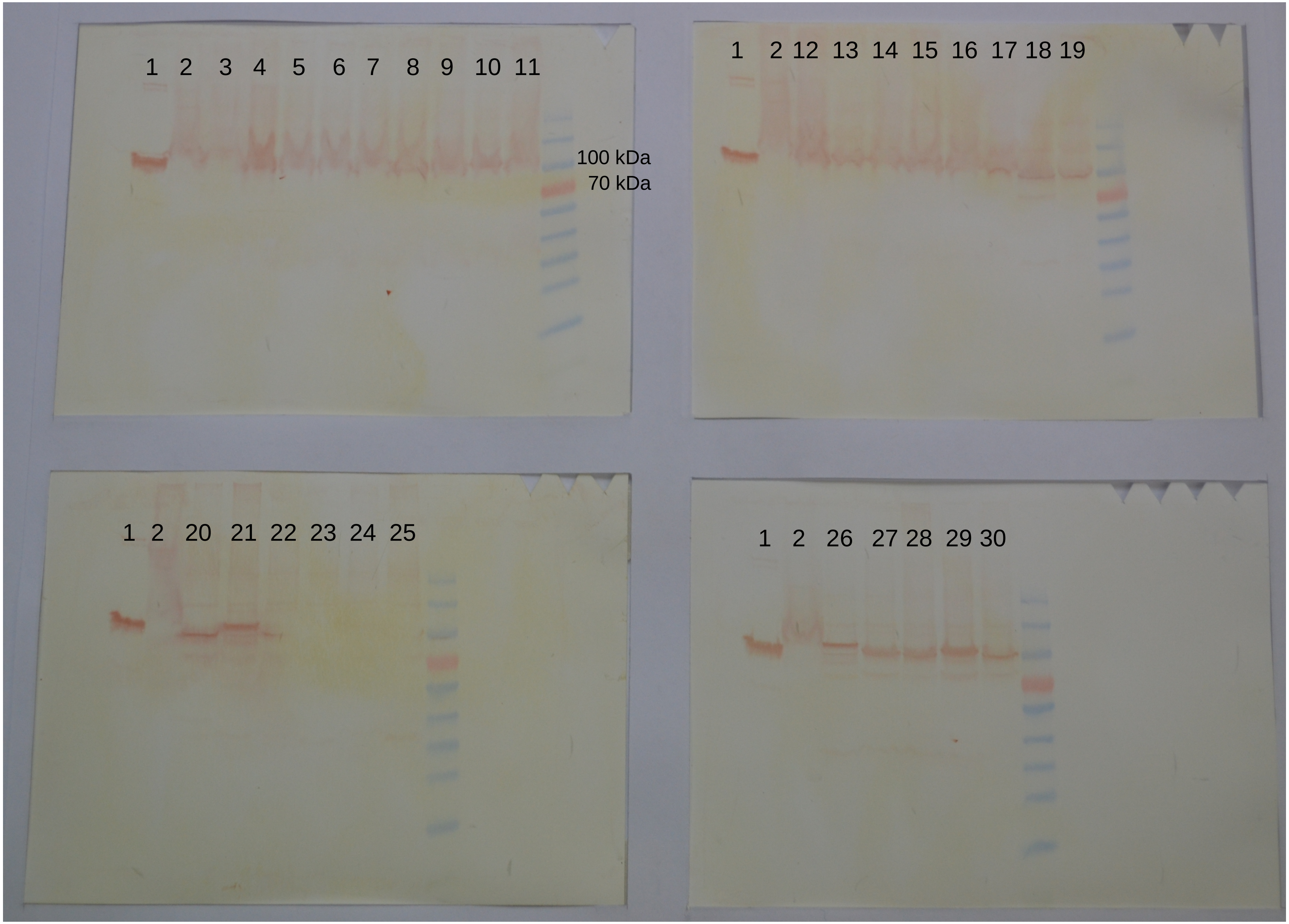

Supplement: Supplementary file 2 [file Image1.pdf]
