# Supplementary figures and images for: Region 4 of Rhizobium etli Primary Sigma Factor (SigA) Confers Transcriptional Laxity in Escherichia coli
Source: Front Microbiol. 2016 Jul 13;7:1078. doi: 10.3389/fmicb.2016.01078 (PMC4943231; doi:10.3389/fmicb.2016.01078)

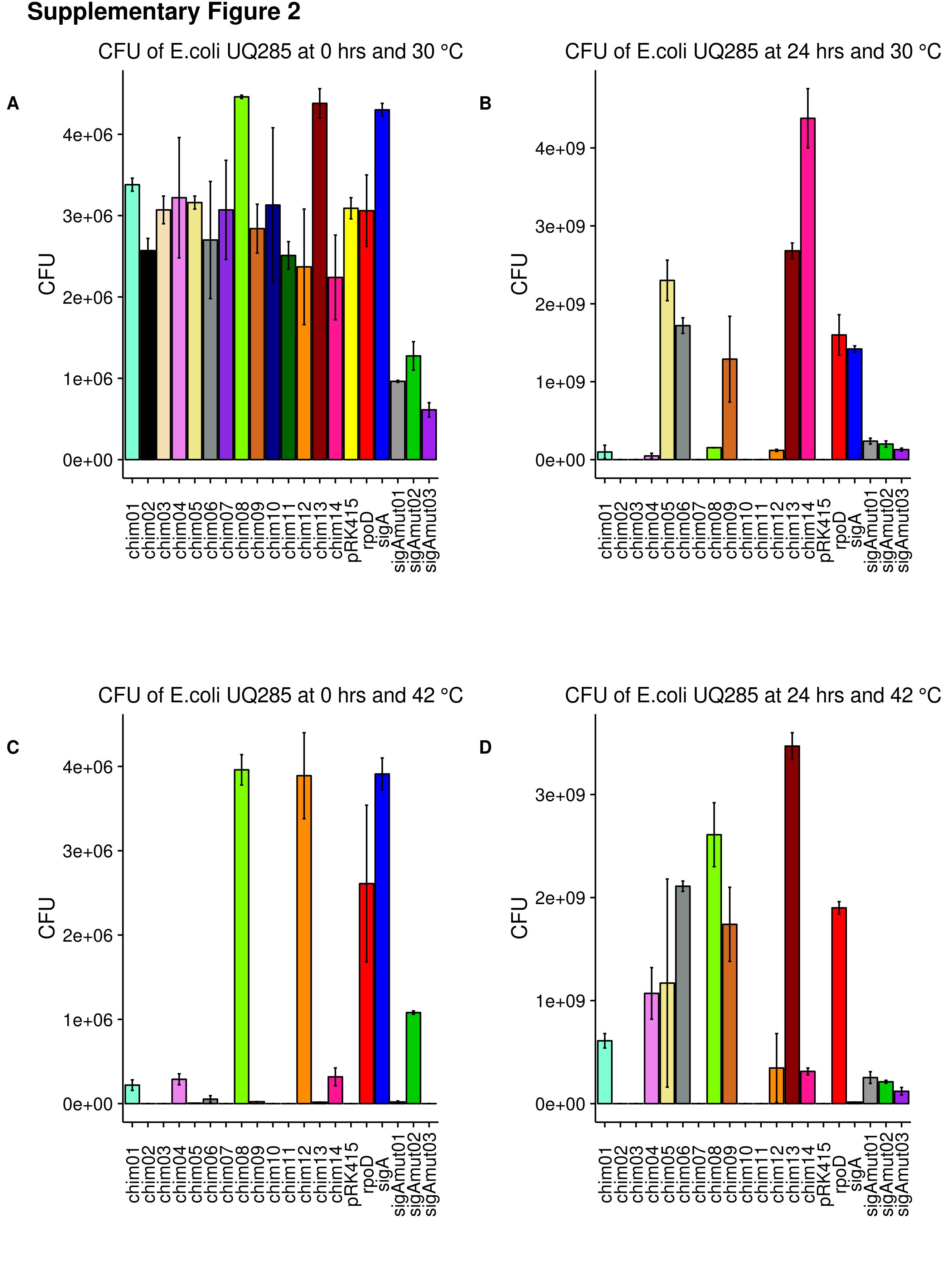

Supplement: Supplementary file 3 [file Image2.JPEG]

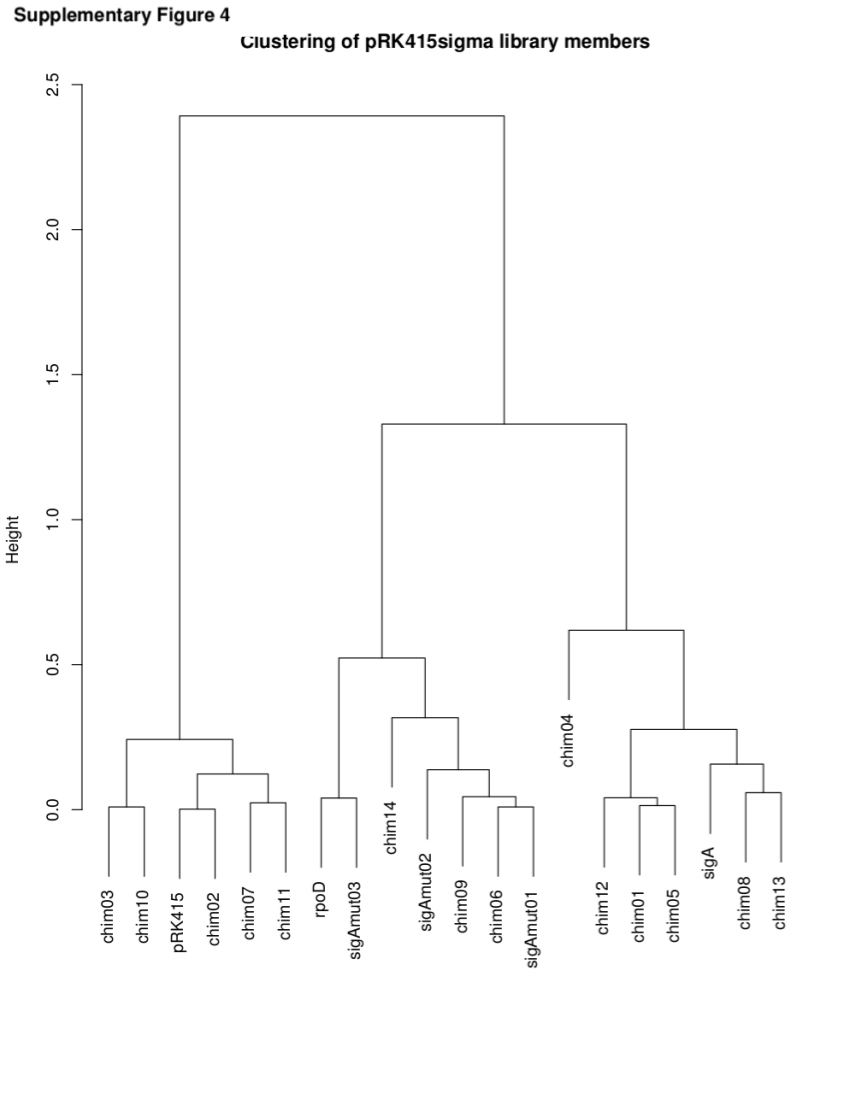

Supplement: Supplementary file 5 [file Image4.jpeg]

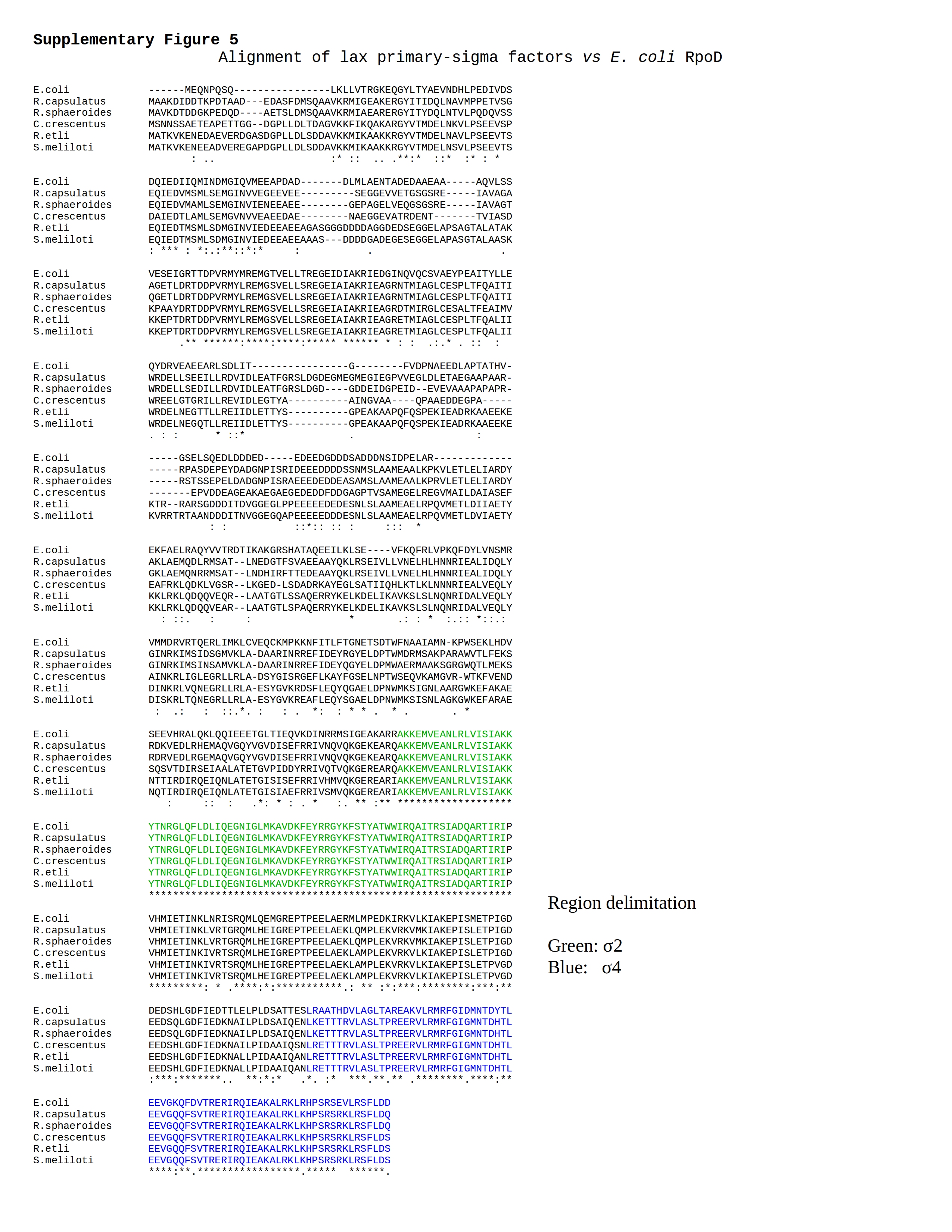

Supplement: Supplementary file 6 [file Image5.jpeg]

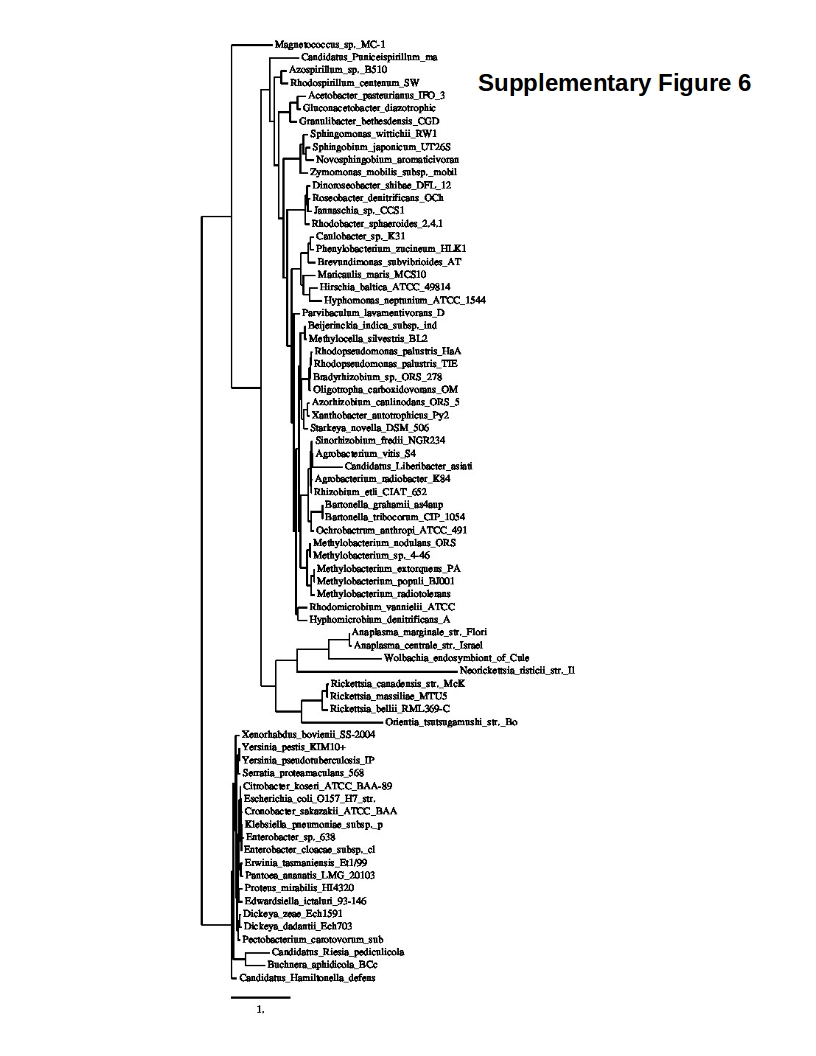

Supplement: Supplementary file 7 [file Image6.jpg]
