# Supplementary material for: Region 4 of Rhizobium etli Primary Sigma Factor (SigA) Confers Transcriptional Laxity in Escherichia coli
Source: Front Microbiol. 2016 Jul 13;7:1078. doi: 10.3389/fmicb.2016.01078 (PMC4943231; doi:10.3389/fmicb.2016.01078)

Supplementary Figure 3A

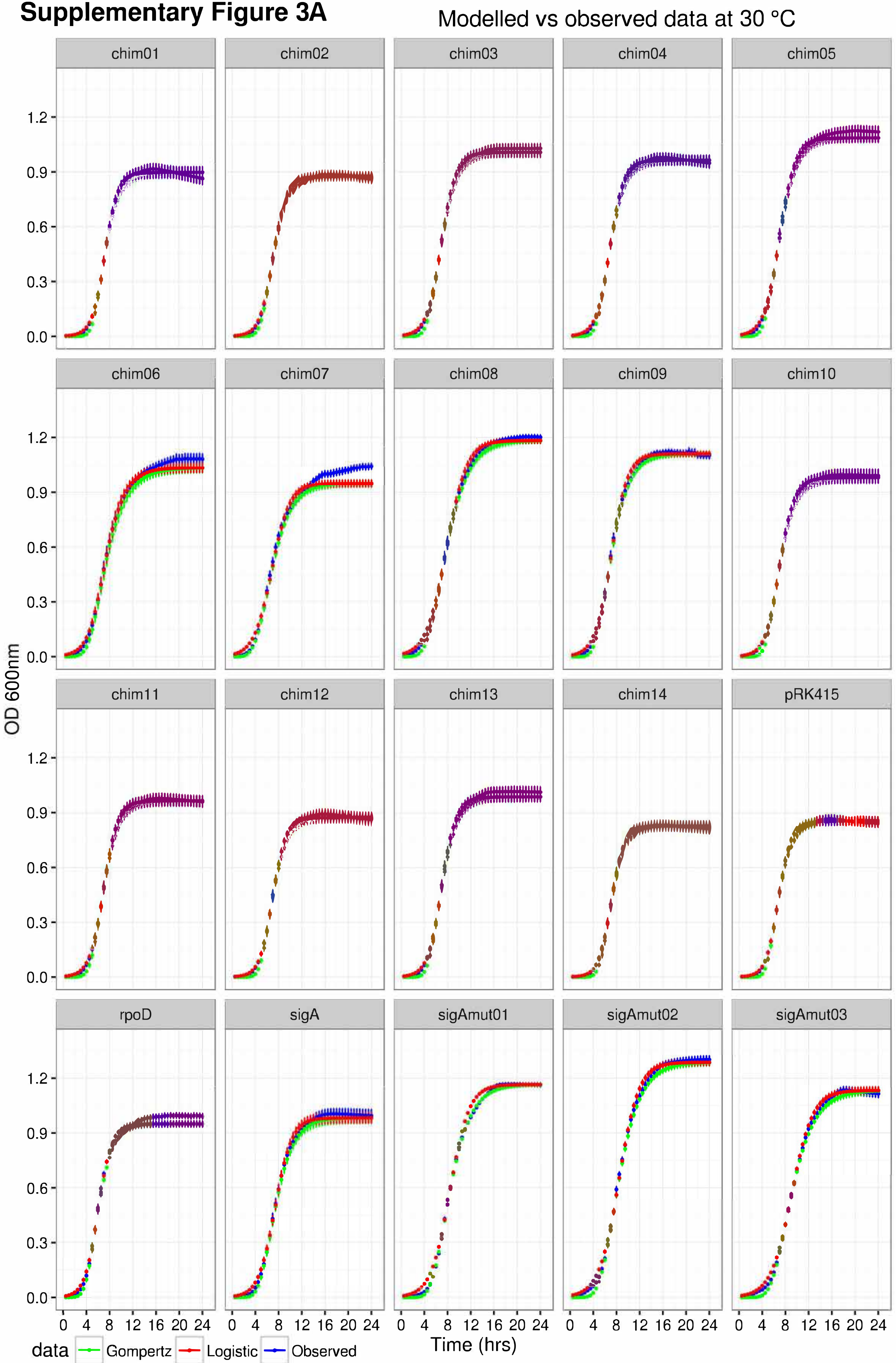

Supplementary Figure 3B

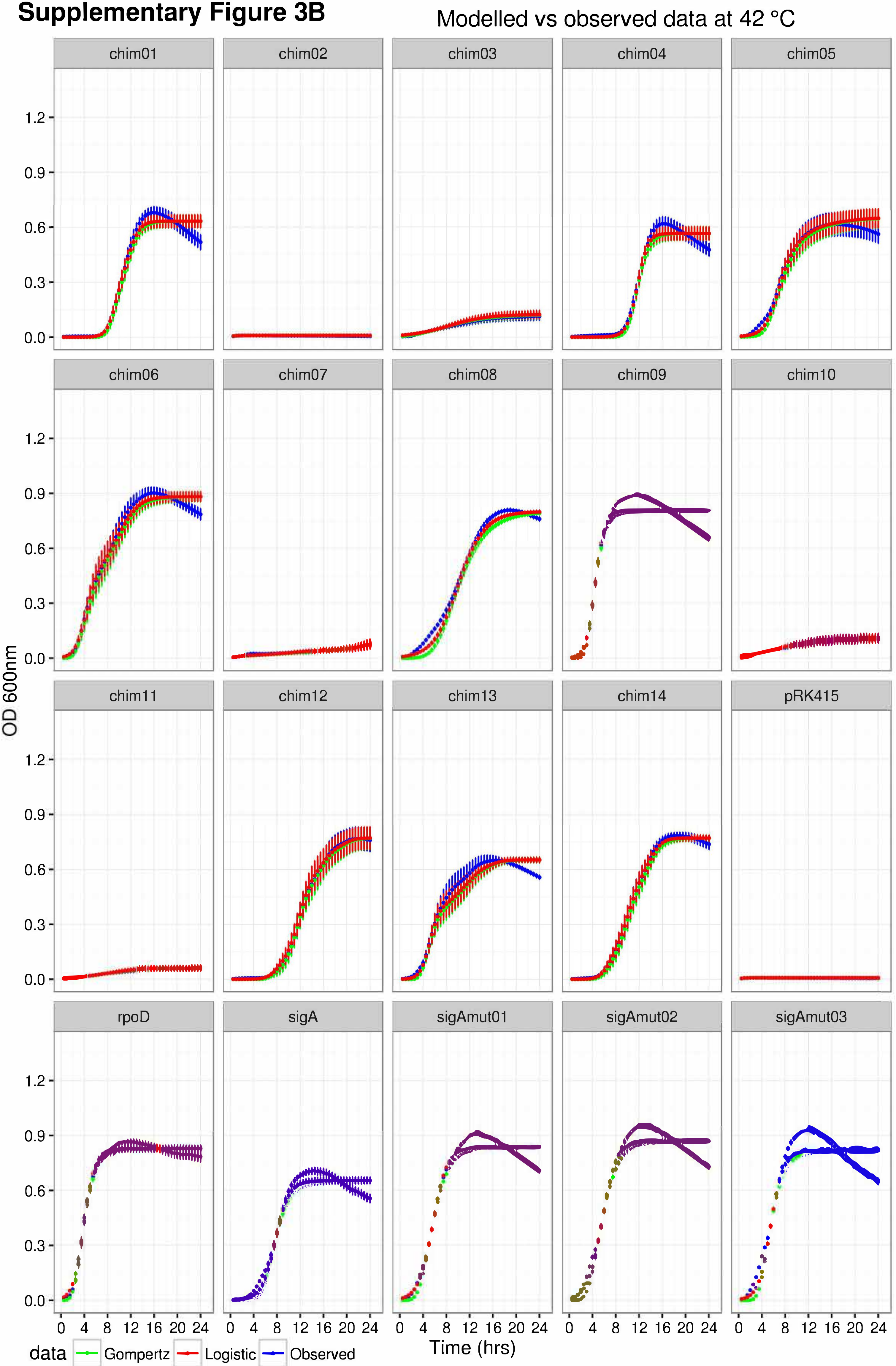

Supplement: Supplementary file 4 [file Image3.pdf]
